# Supplementary material for: Tumor-targeted nanodrug FSGG/siGal-9 for transdermal photothermal immunotherapy of melanoma
Source: Commun Biol. 2024 Feb 16;7:188. doi: 10.1038/s42003-024-05891-6 (PMC10873409; doi:10.1038/s42003-024-05891-6)
Supplement: Supplementary file 2 — Supplementary Information [file 42003_2024_5891_MOESM2_ESM.pdf]

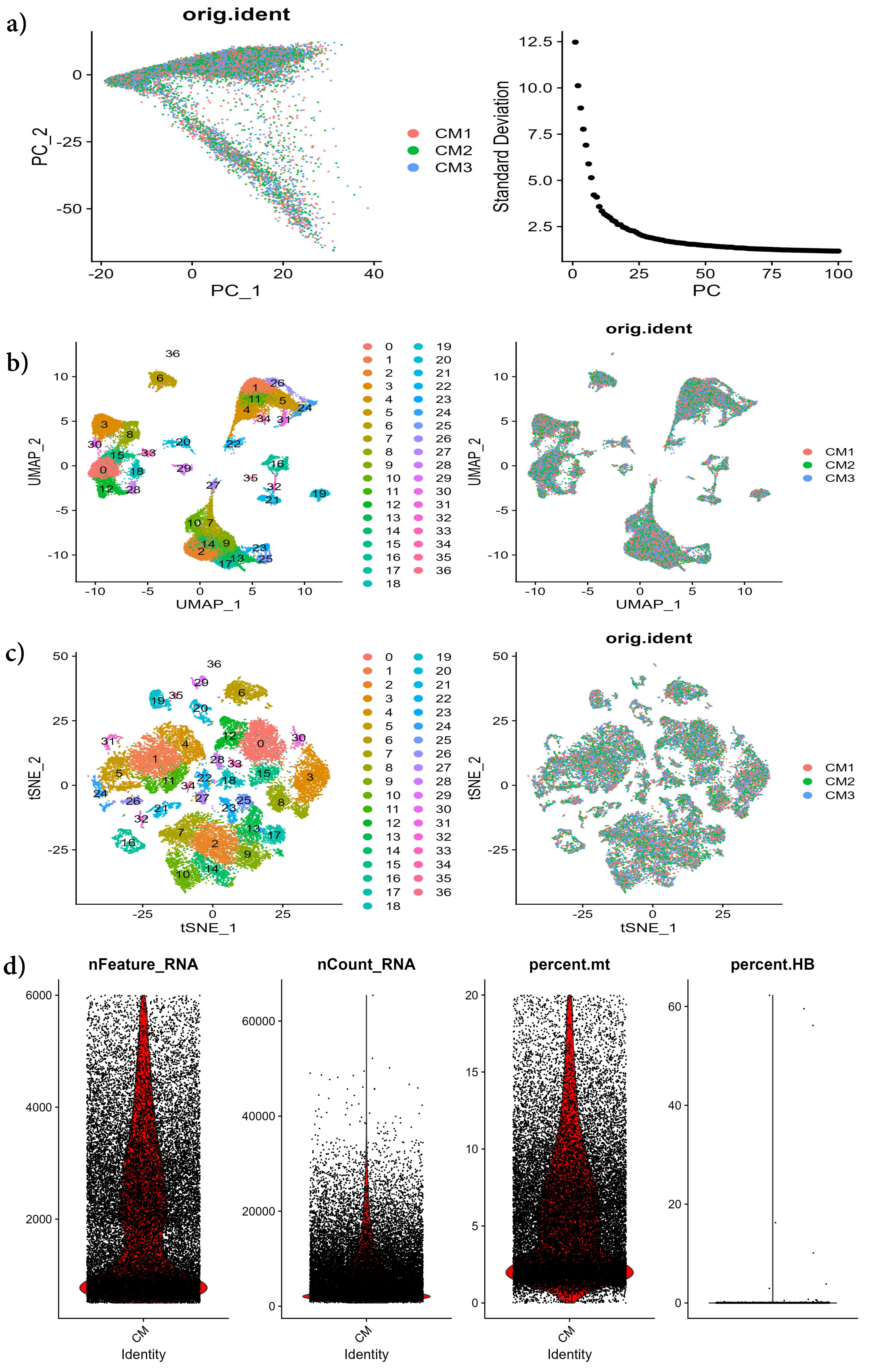

Supplementary Fig. 1: The PCA reduction dimension results a), cells clusters (dims = 70) were shown using UMAP b) and tSNE c), feature RNA numbers, total read counts, and percentages of mitochondrial genes and haemoglobin genes were calculated after quality control d).

LGALS9 **galectin 9 [*Homo sapiens* (human)]**
Gene ID: 3965, updated on 24-Sep-2023

HPA RNA-seq normal tissues

- Project title: HPA RNA-seq normal tissues
- Description: RNA-seq was performed of tissue samples from 95 human individuals representing 27 different tissues in order to determine tissue-specificity of all protein-coding genes
- BioProject: PRJEB4337
- Publication: PMID 24309898
- Analysis date: Wed Apr 4 07:08:55 2018

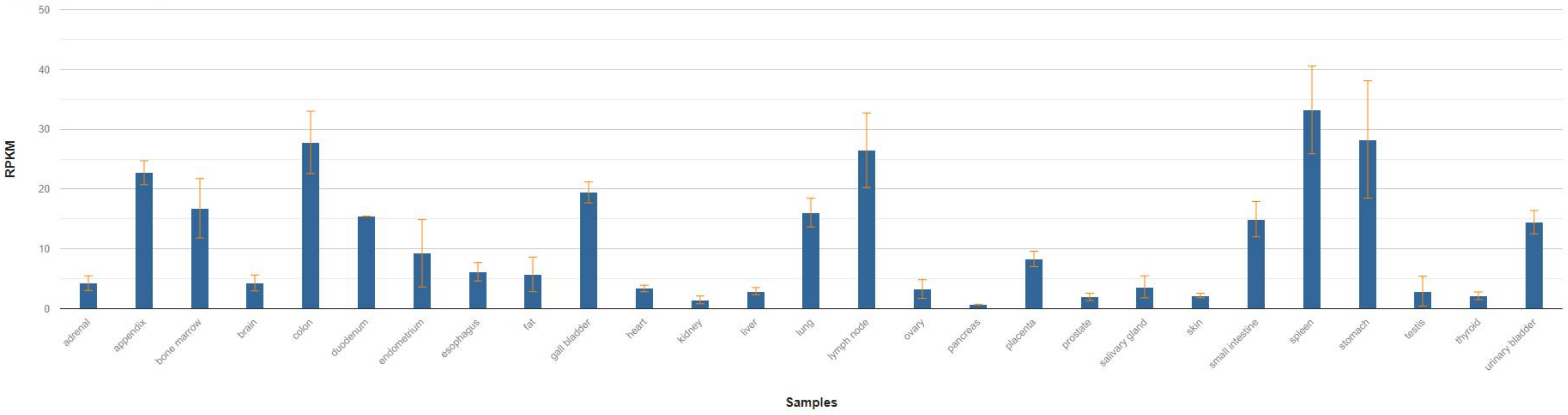

HAVCR2 **hepatitis A virus cellular receptor 2 [*Homo sapiens* (human)]**
Gene ID: 84868, updated on 24-Sep-2023

HPA RNA-seq normal tissues

- Project title: HPA RNA-seq normal tissues
- Description: RNA-seq was performed of tissue samples from 95 human individuals representing 27 different tissues in order to determine tissue-specificity of all protein-coding genes
- BioProject: PRJEB4337
- Publication: PMID 24309898
- Analysis date: Wed Apr 4 07:08:55 2018

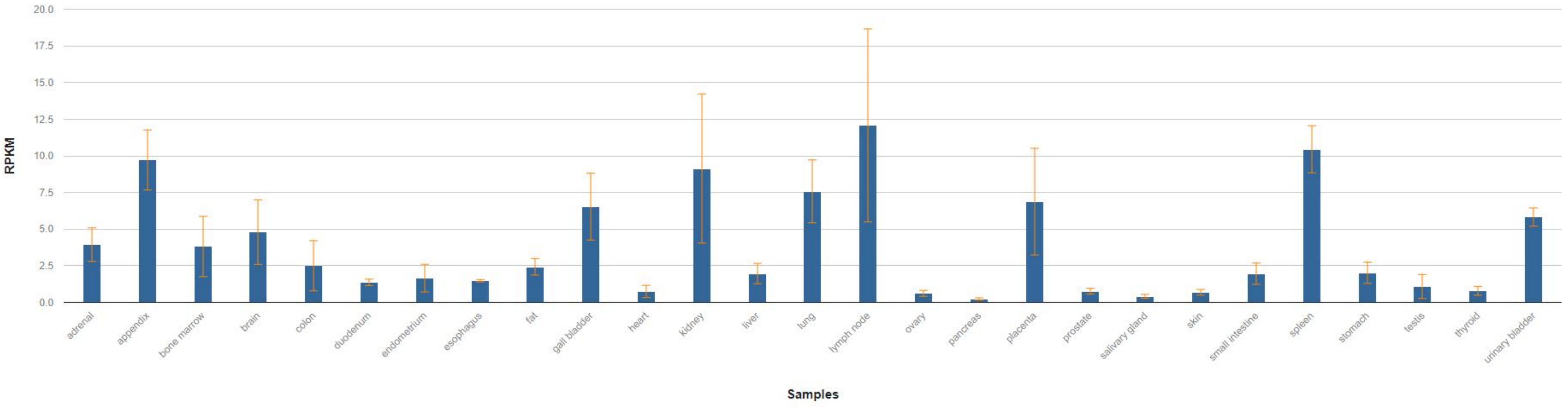

FOXP3 **forkhead box P3 [*Homo sapiens* (human)]**
Gene ID: 50943, updated on 24-Sep-2023

HPA RNA-seq normal tissues

- Project title: HPA RNA-seq normal tissues
- Description: RNA-seq was performed of tissue samples from 95 human individuals representing 27 different tissues in order to determine tissue-specificity of all protein-coding genes
- BioProject: PRJEB4337
- Publication: PMID 24309898
- Analysis date: Wed Apr 4 07:08:55 2018

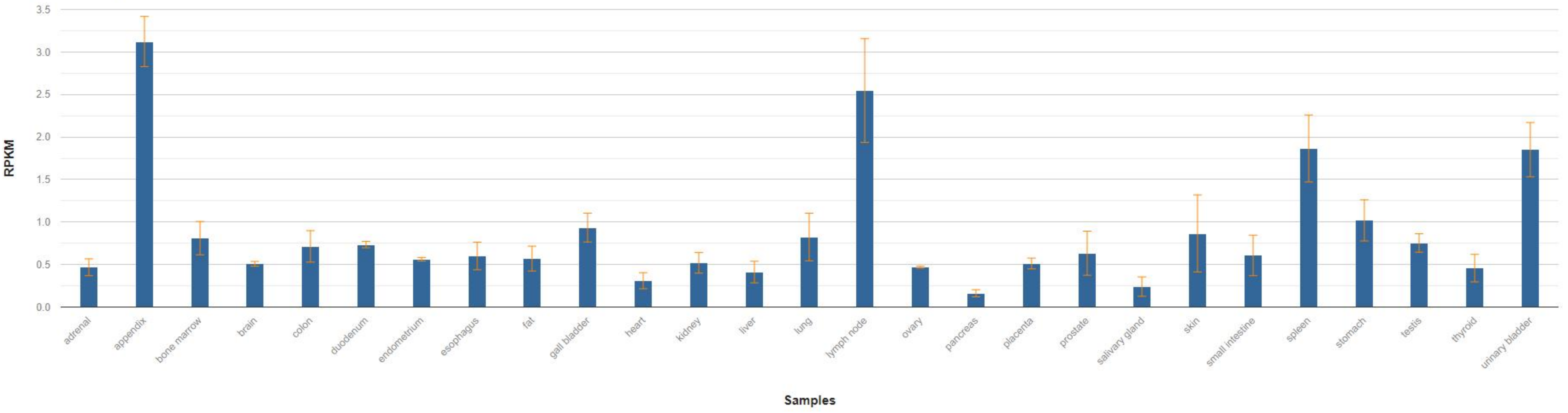

ACTB **actin beta [*Homo sapiens* (human)]**
Gene ID: 60, updated on 7-Sep-2023

HPA RNA-seq normal tissues

- Project title: HPA RNA-seq normal tissues
- Description: RNA-seq was performed of tissue samples from 95 human individuals representing 27 different tissues in order to determine tissue-specificity of all protein-coding genes
- BioProject: PRJEB4337
- Publication: PMID 24309898
- Analysis date: Wed Apr 4 07:08:55 2018

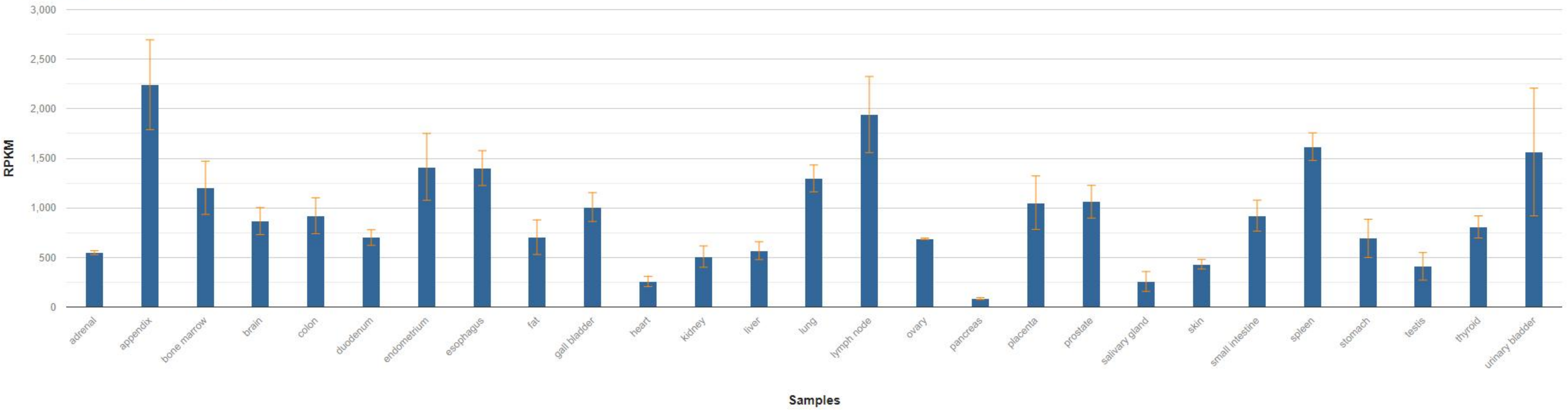

Supplementary Fig. 2: Broad expression levels of Gal-9 (LGALS9), TIM-3 (HAVCR2), FOXP3 and β-actin in human tissues and organs. RNA-seq was performed of tissue samples from 95 human individuals representing 27 different tissues in order to determine tissue-specificity of all protein-coding genes (The bar graphs are available at https://www.ncbi.nlm.nih.gov/gene/, or the primary data (reads) are available at https://www.ebi.ac.uk/biostudies/arrayexpress/studies/E-MTAB-1733?query=E-MTAB-1733).

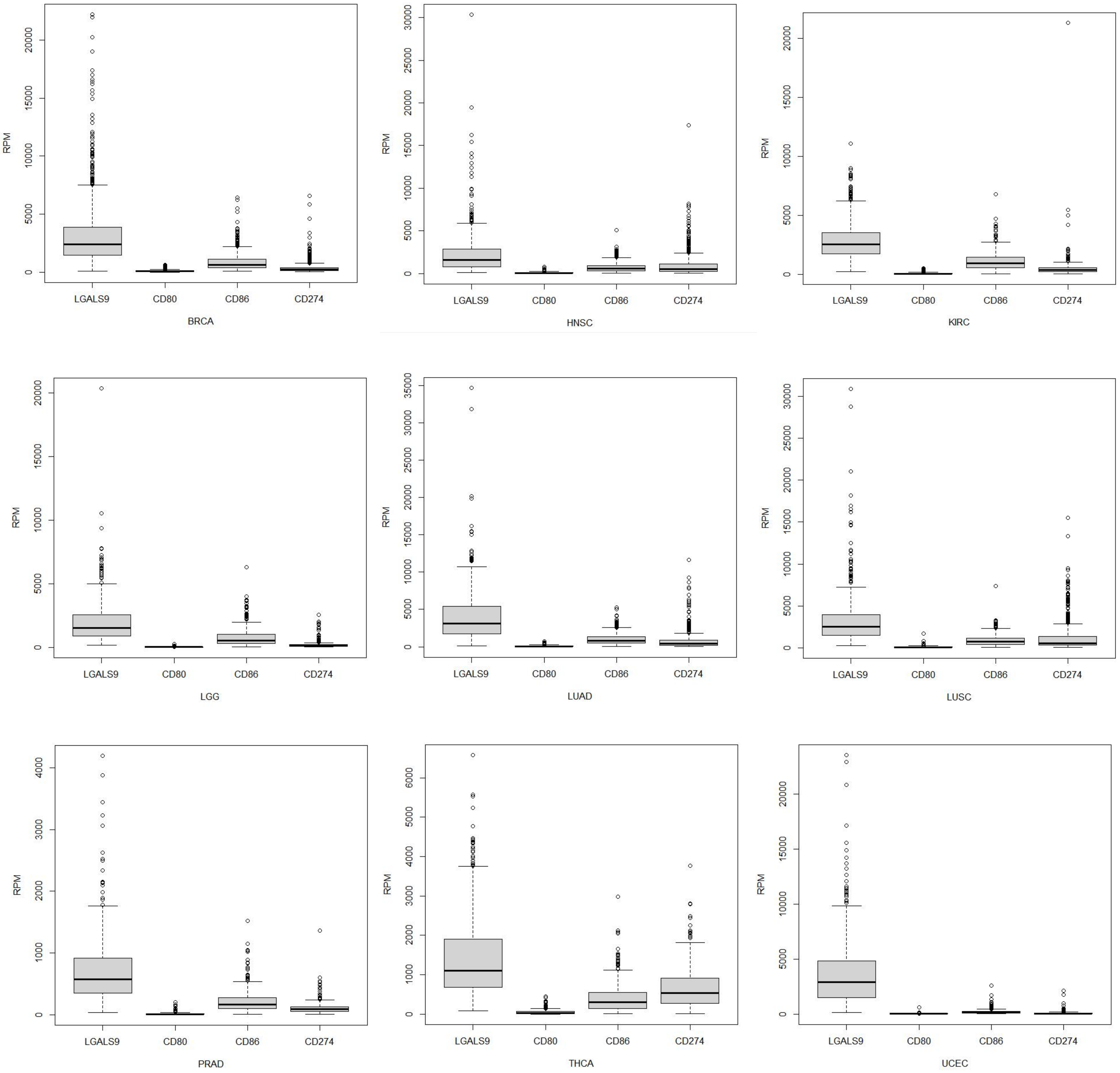

Supplementary Fig. 3: Expression levels of Gal-9 (LGALS9), PD-L1 (CD274) and CD80/86 in the top ten type primary soild tumors from TCGA database (The Cancer Genome Atlas).

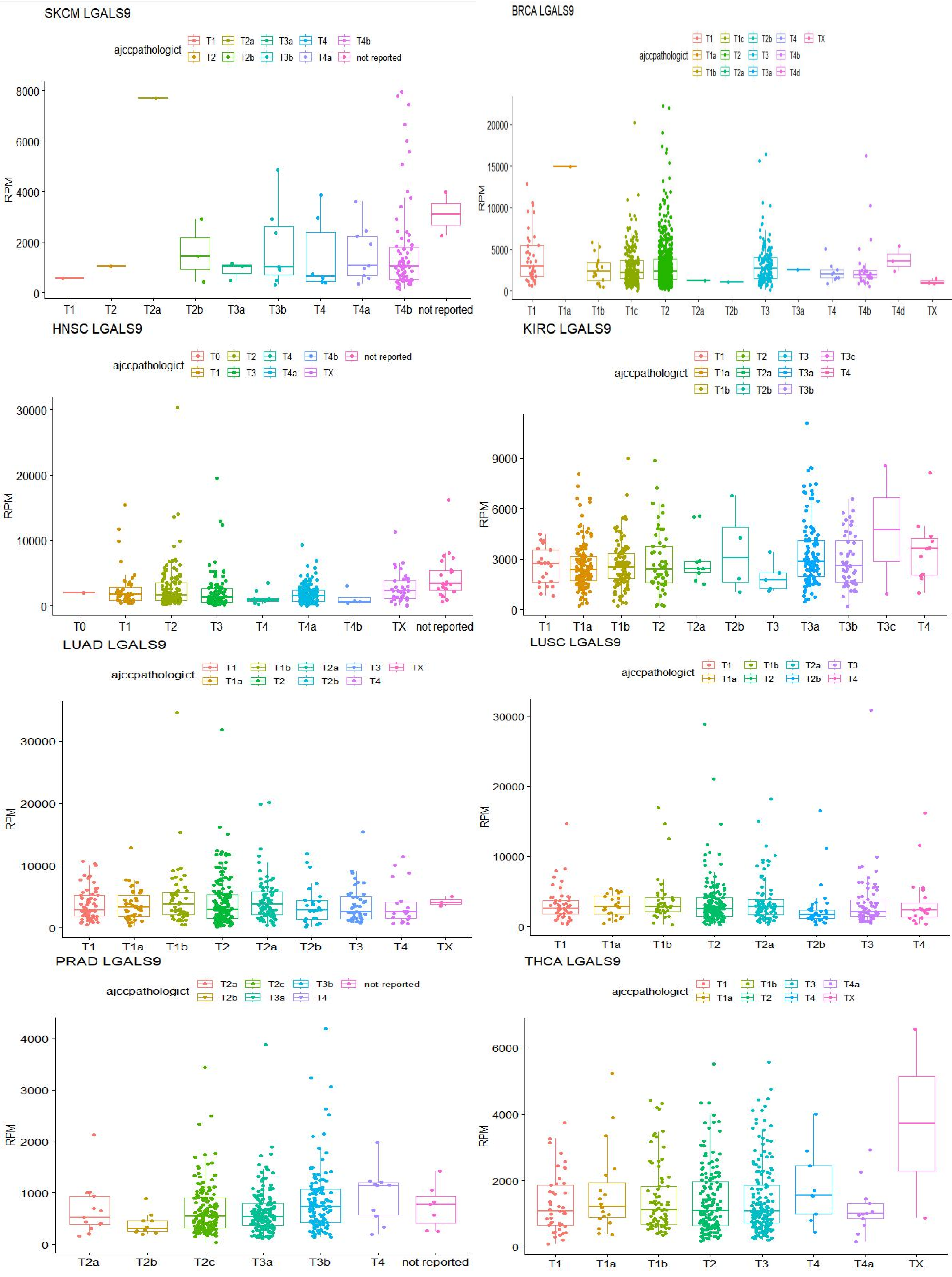

Supplementary Fig. 4: Relationships of LGALS9 (Gal-9) expression with tumor stages (Ajcc_pathologic_t) in the top ten type primary soild tumors from TCGA database (The Cancer Genome Atlas).

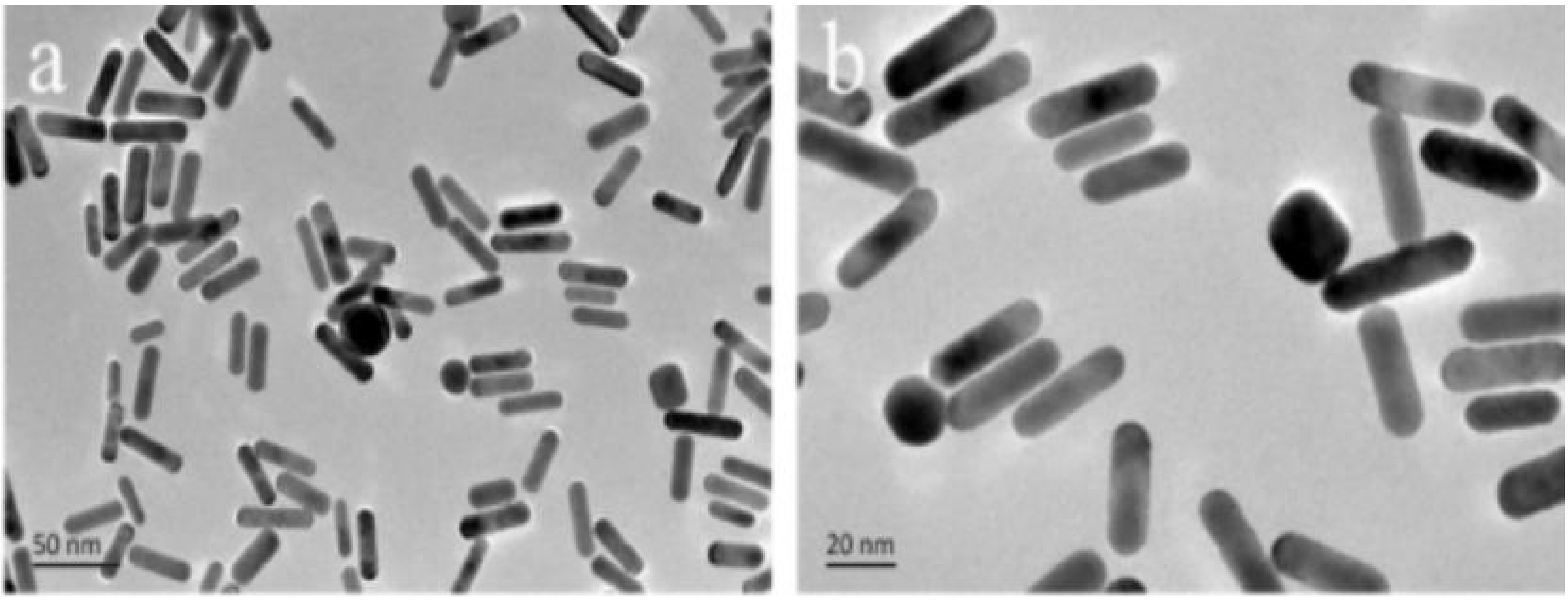

Supplementary Fig. 5: The appearance of GNR-MUA was characterized using TEM.

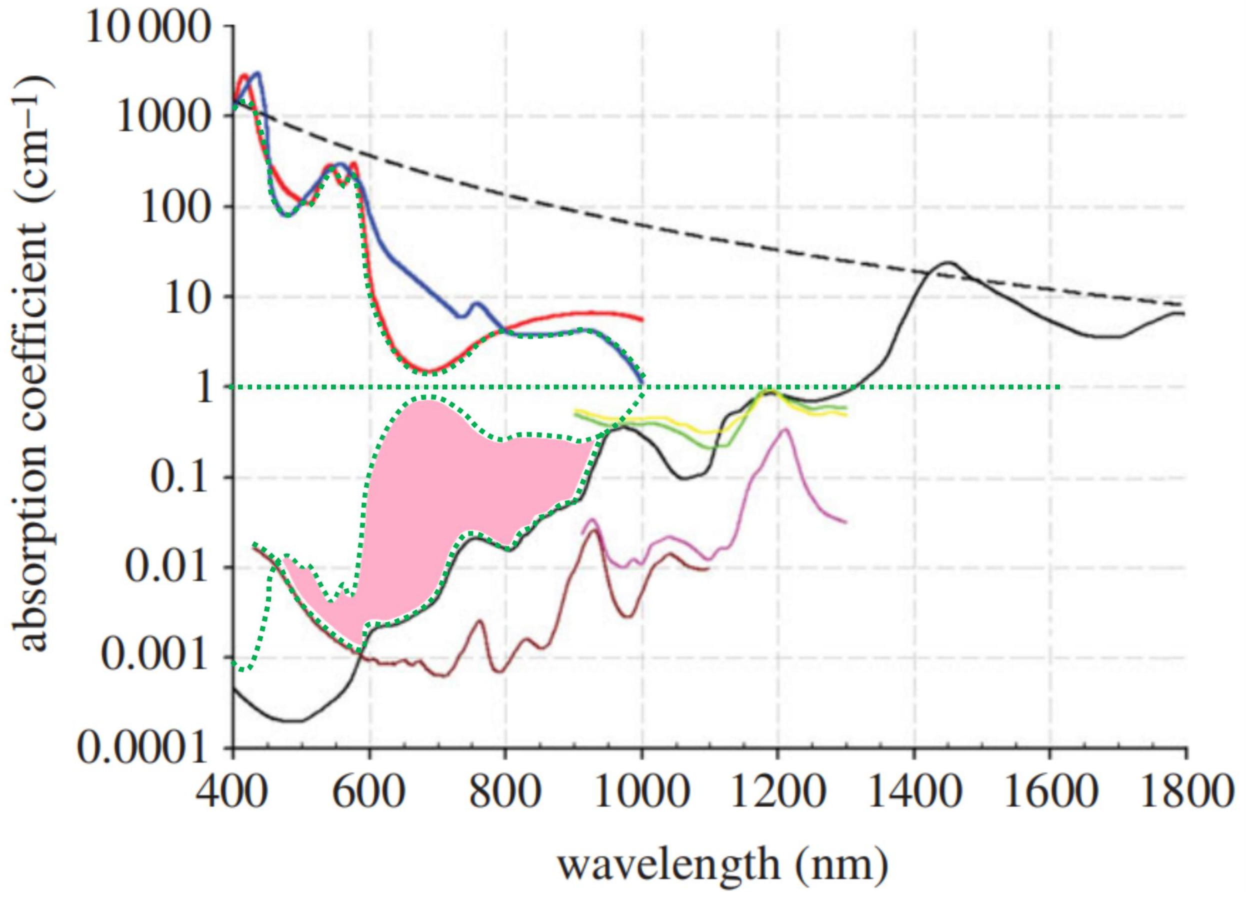

Supplementary Fig. 6: The absorption coefficient (α') of electromagnetic waves by biomacromolecules. Oxyhaemoglobin (HbO_2), red line (150 g/L); deoxyhaemoglobin (HHb), blue line (150 g/L); water, black Line; lipid (a), brown line; lipid (b), pink line; melanin, black dashed line (α' corresponds to that in skin). Collagen (green line) and elastin (yellow line). (Data derived from http://omlc.ogi.edu/spectra/)

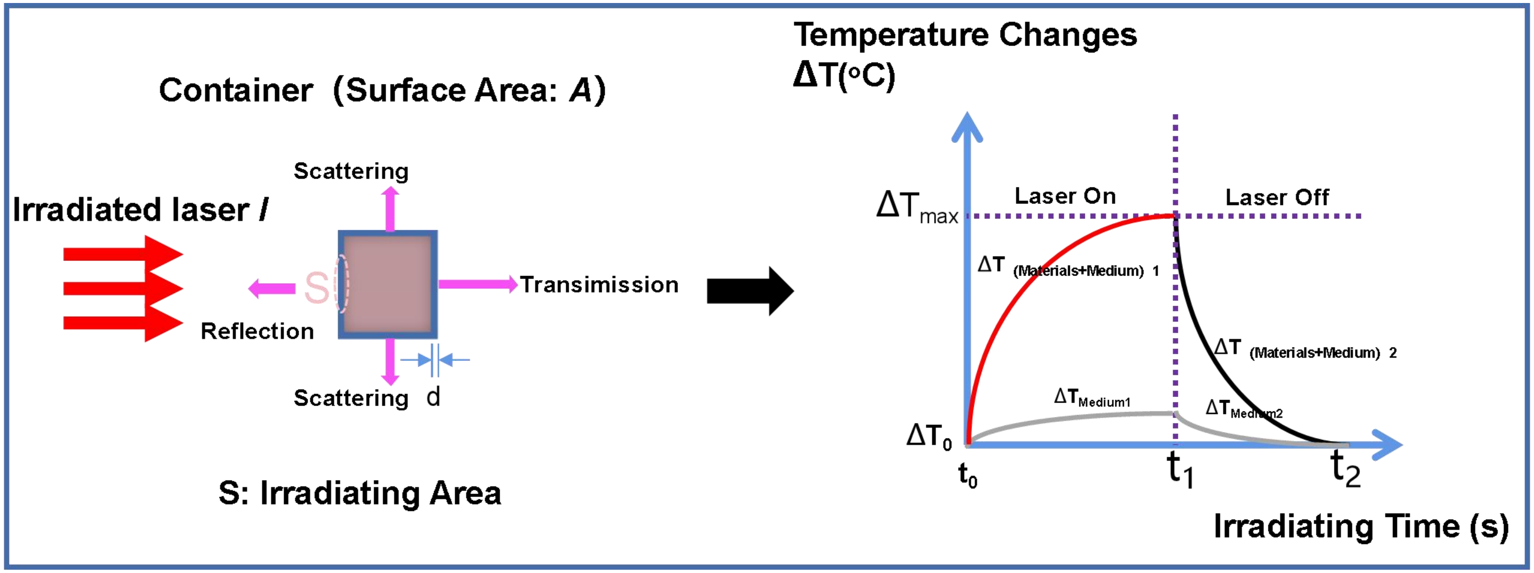

Supplementary Fig. 7: Temperature changes of the system.

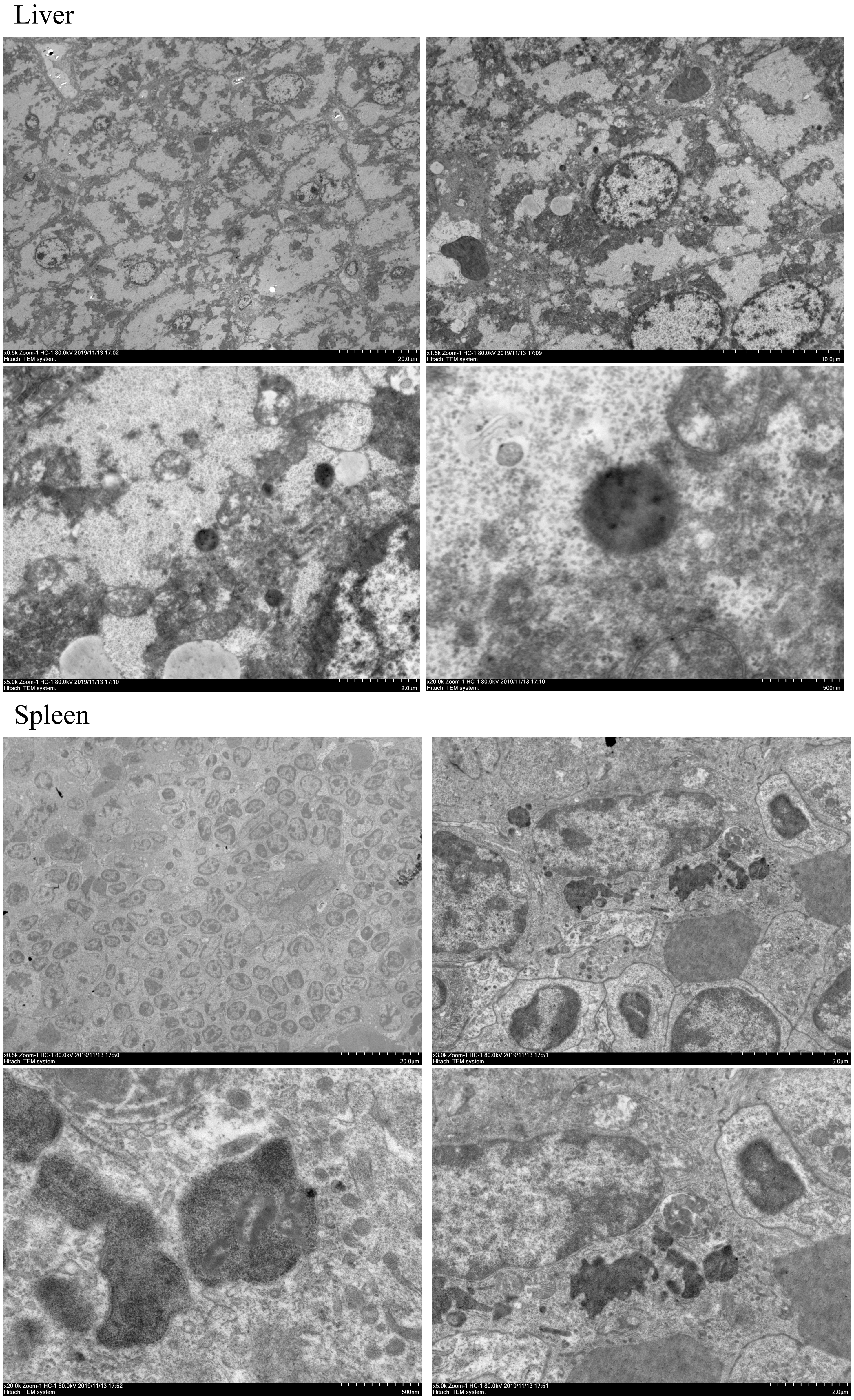

Supplementary Fig. 8: TEM images of liver and spleen from the 22-day PBS-treating mice (Controls).

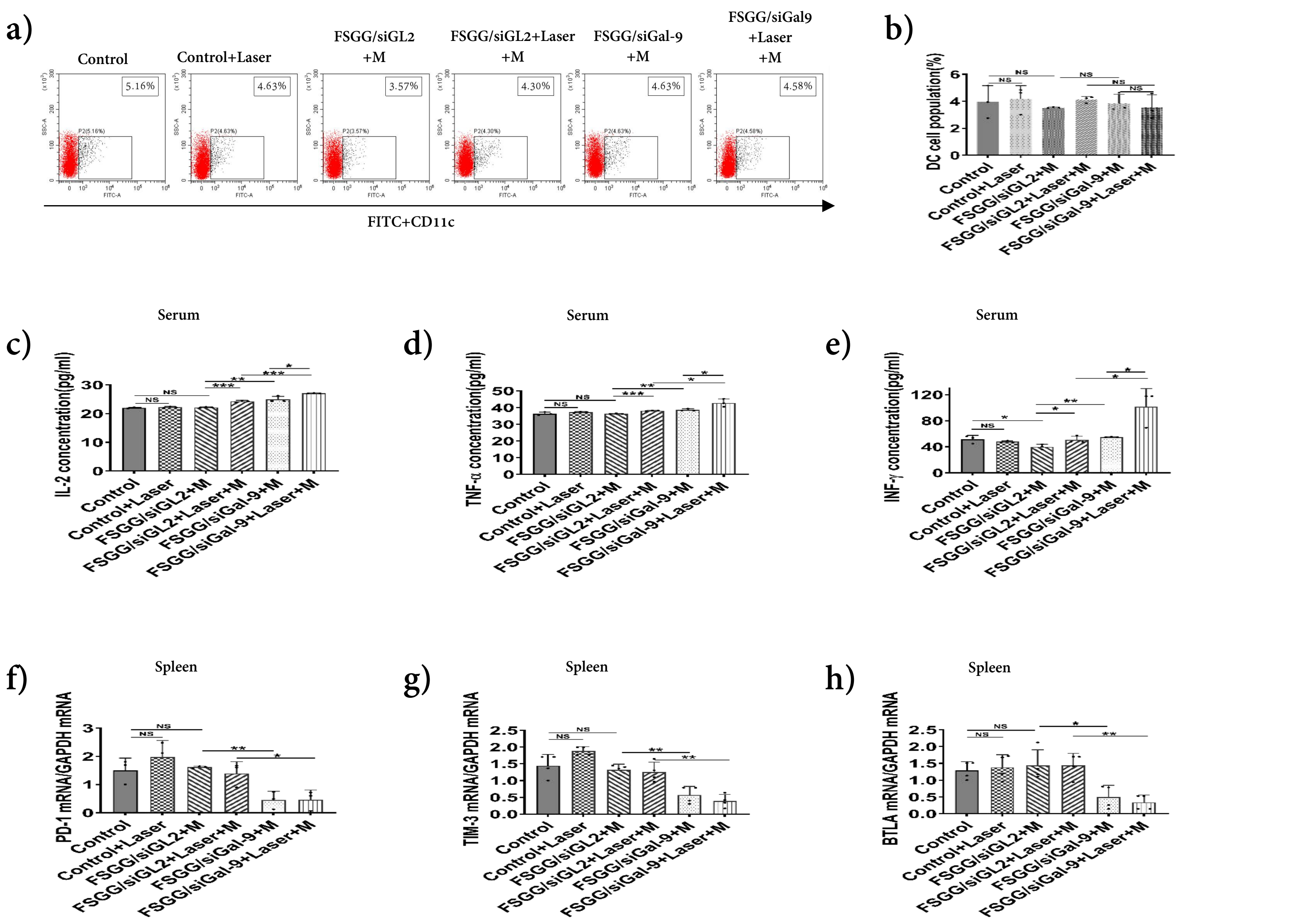

Supplementary Fig. 9: Melanoma-bearing mice were treated as described in Fig. 5(c-f). Mouse splenic cells and sera were collected as described in Materials and Methods. a) and b) Percentages of DCs in splenic cells detected by flow cytometry after CD11c-FITC staining. The percentages of DC population were calculated. c), d) and e) Upregulation of inflammatory cytokines in sera. The levels of IL-2, TNF-α, and IFN-γ were analyzed by ELISA kits (c), (d) and (e). f), g) and h) Reversing splenic T cell exhaustion. The expression levels of PD-1, TIM-3, and BTLA on splenic T cells were analyzed by RT-q-PCR. Error bars represent the standard deviation of three experiments (n.s. P > 0.05; *P ≤ 0.05; **P ≤ 0.01; Adding the magnet field is denoted as “M”).

Supplementary Fig. 10-13: Flow Cytometry Gating Strategy. Percentages of Gal-9+ DCs in splenic cells detected by flow cytometry: blank tube (Supplementary Fig. 8), CD11c-FITC staining tube (Supplementary Fig. 9), Gal9-PE staining tube (Supplementary Fig. 10) and CD11c-FITC and Gal-9-PE double staining tube (Supplementary Fig. 11).

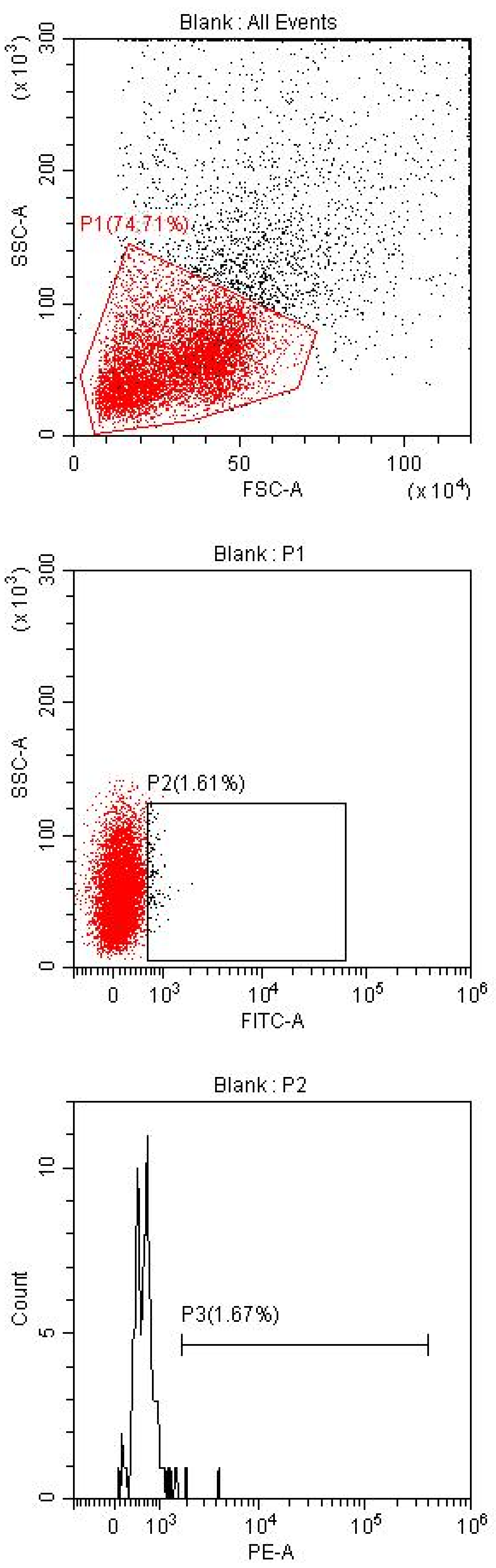

Supplementary Fig. 10

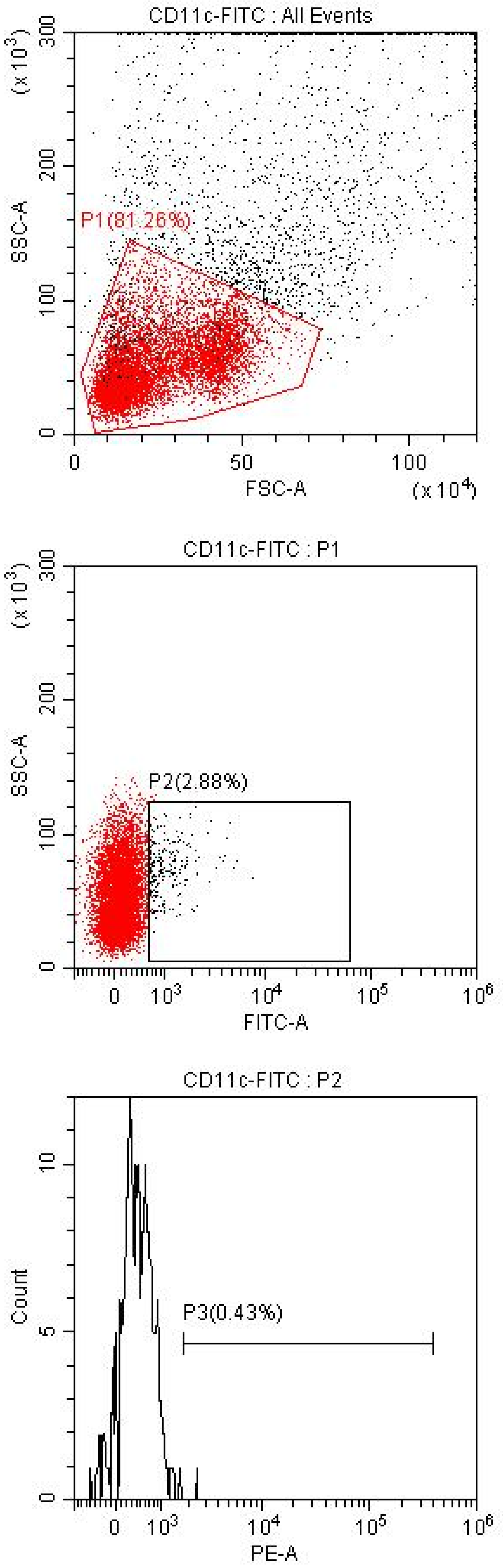

Supplementary Fig. 11

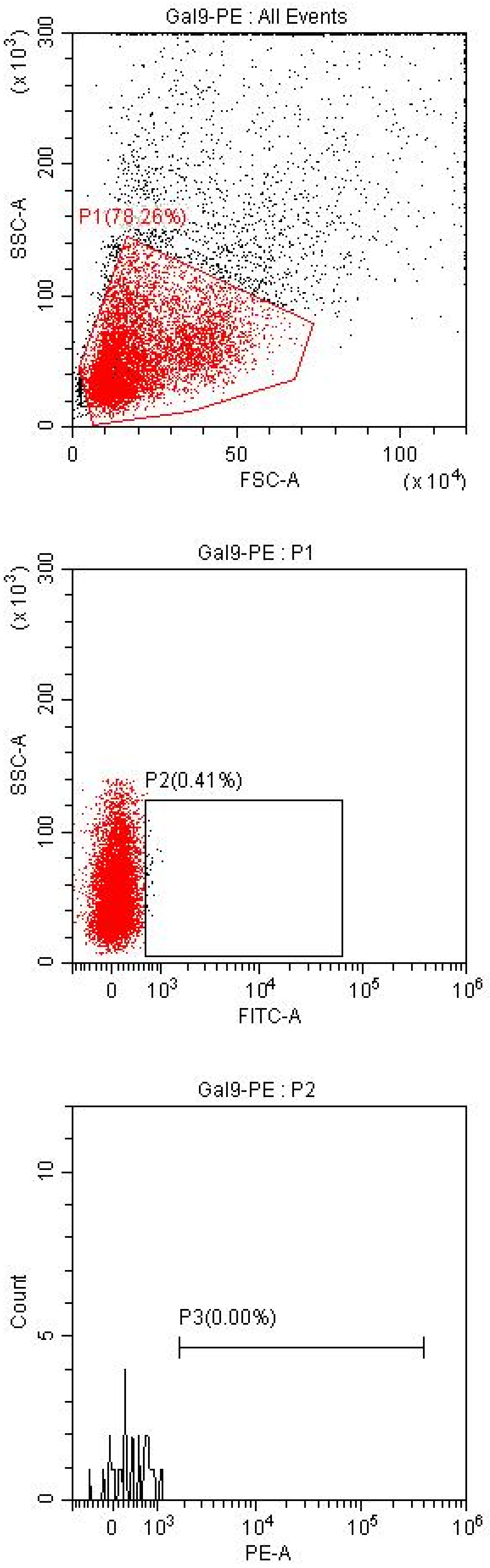

Supplementary Fig. 12

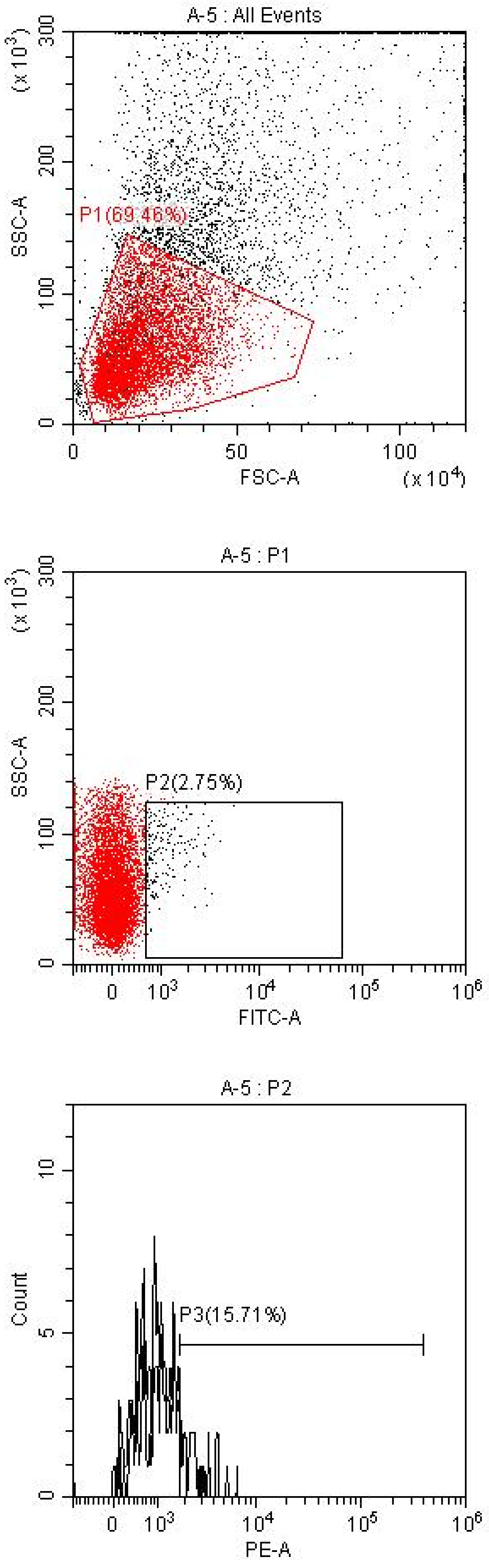

Supplementary Fig. 13

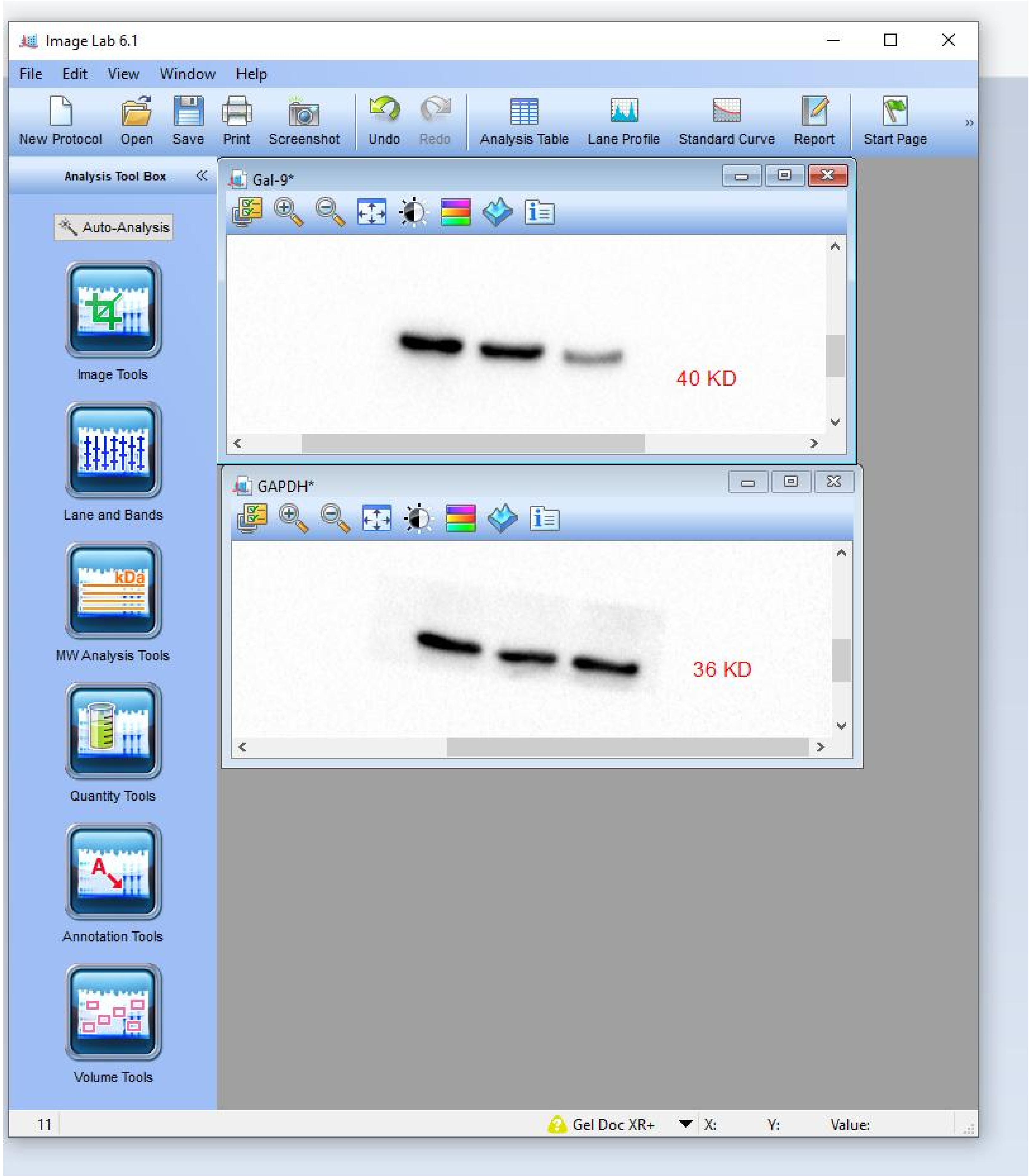

Supplementary Fig. 14: Uncropped and unedited blot/gel images of Gal-9 and GAPDH.

Supplementary Table 1. Significantly higher expressed genes (Tumor v.s. normal tissues; padj <0.01 & log2FoldChange >1) in the top ten type primary soild tumors including HNSC, KIRC, LUAD, LUSC, PRAD, THCA and UCEC from TCGA database (The Cancer Genome Atlas).

		HNSC						KIRC						LUAD						LUSC						PRAD						THCA						UCEC					
Number	Gene	baseMean	log2FoldChange	lfcSE	stat	pvalue	padj	baseMean	log2FoldChange	lfcSE	stat	pvalue	padj	baseMean	log2FoldChange	lfcSE	stat	pvalue	padj	baseMean	log2FoldChange	lfcSE	stat	pvalue	padj	baseMean	log2FoldChange	lfcSE	stat	pvalue	padj	baseMean	log2FoldChange	lfcSE	stat	pvalue	padj	baseMean	log2FoldChange	lfcSE	stat	pvalue	padj
1	ACO78983.1	3.682198724	1.368418039	0.422755757	3.236899833	0.001208358	0.003618495	2.706678597	1.069831989	0.224434197	4.766795795	1.87E-06	4.39E-06	4.4545772	1.21605078	0.335571684	3.623818215	0.000290286	0.000730701	5.803602879	1.938348136	0.287539994	6.741142716	1.57E-11	6.48E-11	2.20645972	1.809389682	0.364165768	4.968588051	6.74E-07	3.63E-06	12.23129028	2.623023874	0.327358477	8.012695733	1.12E-15	1.48E-14	3.147084334	2.662443771	0.498225957	5.343847972	9.10E-08	5.78E-07
2	AC112777.1	48.61522106	1.553683006	0.221912615	7.001328021	2.54E-12	3.26E-11	7.370398626	2.816897532	0.254778346	11.05626746	2.04E-28	1.62E-27	15.88248054	3.650162383	0.283836231	12.86010024	7.55E-38	2.38E-36	41.73305634	4.118833495	0.215185147	19.14088191	1.15E-81	9.44E-80	6.885933656	1.139597347	0.263297925	4.328166844	1.50E-05	6.45E-05	4.834830587	1.681862592	0.296214079	5.677861761	1.36E-08	9.10E-08	21.59113556	4.355865933	0.594931083	7.321631128	2.45E-13	3.30E-12
3	ASF1B	1155.76312	1.312962085	0.202808103	6.473913335	9.55E-11	9.90E-10	278.2456651	2.475501938	0.14357716	17.24161371	1.29E-66	4.58E-65	458.1898319	2.365192767	0.154917103	15.26747351	1.26E-52	8.56E-51	1368.313958	3.344432793	0.152447936	21.93819654	1.12E-106	1.91E-104	216.1838815	1.061941965	0.146621242	7.242756559	4.40E-13	5.78E-12	366.0693139	1.961174312	0.145181102	13.50846829	1.39E-41	1.35E-39	849.7761342	4.711596236	0.270073041	17.44563699	3.72E-68	1.70E-65
4	ASPM	1678.587338	1.624783737	0.24531708	6.623198573	3.52E-11	3.85E-10	268.964165	3.372420654	0.167260881	20.16263833	2.08E-90	1.60E-88	496.5230507	3.448843996	0.209967216	16.42563095	1.25E-60	1.29E-58	1500.456164	4.571548056	0.179200749	25.51076423	1.50E-143	1.21E-140	149.9467141	1.685603411	0.184603238	9.130952583	6.79E-20	2.32E-18	137.4579289	1.29884463	0.222328856	5.841997531	5.16E-09	3.60E-08	471.5536458	4.758826816	0.252399133	18.85437069	2.71E-79	2.20E-76
5	AURKB	1081.939444	1.550627417	0.232606882	6.666300677	2.62E-11	2.92E-10	102.1579719	3.093410391	0.175004734	17.67615268	6.40E-70	2.56E-68	269.5161051	3.209997456	0.202322386	15.86575524	1.09E-56	9.12E-55	984.1705749	4.476135546	0.19249215	23.25360047	1.31E-119	3.31E-117	91.3503568	1.389382985	0.154440831	8.996215403	2.34E-19	7.32E-18	175.4316753	1.639989892	0.246268813	6.659348662	2.75E-11	2.43E-10	533.0081944	5.332482517	0.334327888	15.94985854	2.86E-57	6.89E-55
6	BIRC5	2525.096772	1.686850739	0.225349349	7.485491951	7.13E-14	1.13E-12	282.0725646	2.875920807	0.166554361	17.26716003	8.31E-67	2.96E-65	953.9104556	3.659914301	0.19909509	18.38274513	1.81E-75	4.12E-73	2878.131173	4.882160317	0.198602735	24.58254326	1.94E-133	8.34E-131	258.0604918	1.869158283	0.167440856	11.16309562	6.18E-29	8.56E-27	225.5728229	1.407231732	0.214202184	6.569642302	5.04E-11	4.34E-10	1194.182619	5.365869884	0.300339501	17.86601449	2.17E-71	1.20E-68
7	BUB1	1212.985194	1.638791372	0.21446423	7.6413273	2.15E-14	3.65E-13	238.3225337	2.839514987	0.147741406	19.2194935	2.54E-82	1.54E-80	594.1695508	2.521630407	0.161714945	15.59305733	8.12E-55	6.08E-53	1564.937934	3.541488465	0.148258123	23.8873149	4.15E-126	1.30E-123	178.4288511	1.220538606	0.167288753	7.29599174	2.96E-13	4.00E-12	232.3148139	1.659795478	0.209642228	7.917276468	2.43E-15	3.12E-14	682.2263521	4.617829991	0.247161021	18.68348805	6.75E-78	5.11E-75
8	BUB1B	767.4716559	1.492924422	0.227425471	6.564455681	5.22E-11	5.59E-10	136.4720786	1.468985743	0.117904239	12.45914269	1.25E-35	1.34E-34	294.5139913	3.418014877	0.197594554	17.29812289	4.86E-67	7.12E-65	992.791281	4.630401111	0.184074388	25.15505369	1.24E-139	7.81E-137	131.0428406	1.584107061	0.14960253	10.58877187	3.36E-26	3.07E-24	156.0619623	1.230875675	0.172686354	7.127810889	1.02E-12	1.04E-11	450.8331752	4.731142806	0.355715414	13.30035926	2.30E-40	2.51E-38
9	CAMK2N2	69.87401718	2.292396821	0.170904786	13.41329795	5.05E-41	1.68E-38	39.90809578	1.478026123	0.17628366	8.384362594	5.10E-17	2.35E-16	36.96536248	2.81929746	0.244976423	11.50844406	1.20E-30	2.55E-29	132.3971364	3.430648351	0.202562648	16.9362337	2.43E-64	1.11E-62	86.36438049	1.070231709	0.170398761	6.280748201	3.37E-10	2.99E-09	69.45900084	3.095341475	0.183329652	16.88401983	5.90E-64	2.65E-61	48.86291905	2.650193538	0.37480989	7.07067358	1.54E-12	1.90E-11
10	CCR8	31.31973664	1.617137215	0.234887783	6.884722556	5.79E-12	7.09E-11	15.80666543	4.035239936	0.330142866	12.22270826	2.35E-34	2.39E-33	25.78060564	1.854926455	0.199378292	9.303552727	1.36E-20	1.45E-19	45.20562103	1.024232903	0.247305435	4.141570539	3.45E-05	8.52E-05	9.66105434	1.088554141	0.224097497	4.857502462	1.19E-06	6.13E-06	16.20488445	1.308124604	0.316160927	4.137527739	3.51E-05	0.000155268	4.24319078	2.646265301	0.460016578	5.752543343	8.79E-09	6.53E-08
11	CDC25C	172.7232158	1.425253055	0.216780483	6.574637327	4.88E-11	5.25E-10	46.16436653	3.306508898	0.194936902	16.9619444	1.57E-64	5.16E-63	105.8650376	3.710897729	0.196677901	18.86789372	2.09E-79	5.61E-77	240.3385379	4.367375386	0.210805464	20.71756259	2.41E-95	2.99E-93	45.80206896	1.942575676	0.176524857	11.0045447	3.63E-28	4.46E-26	27.34846124	1.061168056	0.197145381	5.382667597	7.34E-08	4.51E-07	144.9868327	5.032963757	0.297386584	16.92397715	3.00E-64	1.13E-61
12	CDC45	764.11053	1.606872505	0.216255419	7.4304381	1.08E-13	1.67E-12	90.62461067	2.811133861	0.159169937	17.6612111	8.34E-70	3.30E-68	229.2575014	2.914309555	0.192100031	15.17079168	5.52E-52	3.63E-50	1013.546529	4.638740025	0.176856482	26.22883807	1.25E-151	1.47E-148	88.45331864	1.218878361	0.170324723	7.156203393	8.29E-13	1.05E-11	125.0589875	1.993641445	0.178375509	11.17665453	5.31E-29	2.05E-27	315.3600805	4.953452476	0.289275445	17.12365346	9.89E-66	3.93E-63
13	CDKN2A	986.7080548	2.165188267	0.357656211	6.053825436	1.41E-09	1.23E-08	185.1203064	5.422170733	0.204934413	26.45807818	2.95E-154	9.29E-152	412.9188038	2.543886889	0.244238333	10.4155924	2.10E-25	3.16E-24	1214.784902	3.416573794	0.297933929	11.46755527	1.92E-30	2.23E-29	108.6505599	1.122808741	0.181171073	6.19750562	5.74E-10	4.93E-09	218.8324465	2.975244324	0.222313339	13.38311202	7.59E-41	6.91E-39	463.4762663	3.565520931	0.35654625	10.00016388	1.52E-23	5.78E-22
14	CENPA	375.3129972	1.867639427	0.229283332	8.145552526	3.78E-16	8.03E-15	48.91383737	2.931412336	0.185820935	15.77546865	4.59E-56	1.13E-54	144.739493	3.493767006	0.211544552	16.51551396	2.84E-61	3.03E-59	479.4584249	4.949380725	0.190522717	25.97790335	8.80E-149	8.49E-146	48.62438712	2.007218169	0.209047678	9.601724305	7.86E-22	3.61E-20	43.27504297	1.122525359	0.219139442	5.122425017	3.02E-07	1.72E-06	217.8208522	5.316073219	0.262548015	20.24800388	3.70E-91	9.27E-88
15	CENPF	3750.439775	1.773072865	0.2184618	8.116168899	4.81E-16	1.01E-14	579.4166624	2.35124214	0.125707101	18.7041314	4.58E-78	2.41E-76	1356.348185	3.341341025	0.19710884	16.95175636	1.87E-64	2.31E-62	3966.906056	4.506451	0.176324543	25.55770703	4.51E-144	3.71E-141	484.9712384	1.776781594	0.172877442	10.27769483	8.88E-25	6.39E-23	350.9065872	1.118708014	0.171169441	6.535676055	6.33E-11	5.41E-10	1975.888681	4.415800192	0.221095525	19.97236348	9.58E-89	1.64E-85
16	CLSPN	577.7542421	1.931228873	0.20158283	9.580324238	9.67E-22	4.11E-20	132.4106287	2.225058625	0.116745896	19.05898796	5.53E-81	3.25E-79	299.4220453	2.819482625	0.197671401	14.26348276	3.70E-46	1.78E-44	667.1737092	3.3521869	0.179887612	18.63489579	1.67E-77	1.20E-75	75.82859775	1.041956321	0.165286787	6.303929934	2.90E-10	2.60E-09	119.4727815	1.663226238	0.166830282	9.969570399	2.07E-23	5.21E-22	307.984472	4.641640644	0.284987762	16.28715776	1.22E-59	3.51E-57
17	CTHRC1	1617.241163	3.687127799	0.300842278	12.25601609	1.56E-34	2.57E-32	922.8058641	3.072343985	0.194923502	15.76179348	5.70E-56	1.40E-54	1713.12213	3.955174439	0.217473884	18.18689387	6.56E-74	1.40E-71	3196.652037	3.5254364	0.215780727	16.33805044	5.29E-60	2.04E-58	187.2391038	1.159256056	0.1974521	5.87107484	4.33E-09	3.26E-08	650.6156396	1.241592943	0.235057766	5.282075831	1.28E-07	7.63E-07	752.4681436	2.580222734	0.355241289	7.263296283	3.78E-13	4.98E-12
18	DLGAP5	1181.954572	1.64746544	0.246527321	6.682689095	2.35E-11	2.63E-10	127.3538349	3.130880055	0.173845121	18.00959401	1.64E-72	7.15E-71	388.6403999	3.684540286	0.207911638	17.72166448	2.85E-70	5.18E-68	1155.807886	4.733119773	0.19214462	24.63311111	5.58E-134	2.48E-131	107.8096488	2.008892139	0.181848959	11.04703679	2.27E-28	2.88E-26	115.7420807	1.633894775	0.245884524	6.644967921	3.03E-11	2.67E-10	489.2550683	5.252716588	0.310589765	16.91207238	3.67E-64	1.34E-61
19	DMBX1	24.38964698	2.236520493	0.459174313	4.870743917	1.11E-06	6.05E-06	20.65898507	1.627783969	0.553303726	2.941935669	0.003261677	0.005548074	25.20819946	4.996913855	0.411995244	12.12857171	7.45E-34	1.92E-32	57.57810668	4.834447926	0.381540822	12.67085369	8.58E-37	1.34E-35	3.813844818	1.814152709	0.354016709	5.124483284	2.98E-07	1.70E-06	140.9490792	6.156275549	0.414340061	14.85802637	6.17E-50	1.06E-47	70.30214252	6.173346719	0.569728749	10.83558928	2.33E-27	1.18E-25
20	DUXAP10	50.54274778	3.644513635	0.335444902	10.86471612	1.70E-27	1.32E-25	28.94236396	2.315691494	0.19402195	11.93520367	7.76E-33	7.41E-32	39.34130606	1.71087708	0.230228894	7.431200527	1.08E-13	6.83E-13	66.60068984	2.950892214	0.220056781	13.40968547	5.31E-41	9.87E-40	122.8952774	1.02003093	0.216386155	4.713938042	2.43E-06	1.19E-05	14.92095151	2.256348819	0.230559212	9.786417995	1.29E-22	3.02E-21	41.15819934	2.56402454	0.438535259	5.846792218	5.01E-09	3.87E-08
21	FAM111B	776.8226364	1.052345194	0.200675224	5.244021528	1.57E-07	9.91E-07	177.3766896	2.953482681	0.161891738	18.24356649	2.33E-74	1.07E-72	301.244963	2.764039807	0.192353305	14.36959872	8.03E-47	4.06E-45	781.2240814	3.391838625	0.20874085	16.24904098	2.27E-59	8.55E-58	254.4976882	1.674794418	0.192980608	8.678563268	4.01E-18	1.08E-16	277.4781623	2.825768208	0.194429816	14.53361562	7.42E-48	1.11E-45	236.4443773	4.651091141	0.372348293	12.49123798	8.33E-36	6.98E-34

22	GPRIN1	700.7674349	2.427184881	0.195856959	12.39264049	2.86E-35	5.14E-33	220.3401756	2.351414176	0.138702368	16.95294906	1.83E-64	6.00E-63	271.8825084	2.900778064	0.159831299	18.14899885	1.31E-73	2.74E-71	546.1799647	2.812666862	0.177587201	15.83822959	1.70E-56	5.68E-55	87.97749374	1.190437022	0.141485166	8.413864527	3.97E-17	9.25E-16	101.0639375	2.457521234	0.164756746	14.9160583	2.59E-50	4.50E-48	188.1210325	2.710763115	0.30206142	8.974211659	2.85E-19	7.36E-18
23	GTSE1	673.877912	1.728842608	0.213897817	8.082563107	6.34E-16	1.31E-14	130.187066	2.678622549	0.145414658	18.42058148	8.98E-76	4.34E-74	255.3040293	3.096453836	0.208581507	14.84529421	7.46E-50	4.43E-48	771.9818323	4.174731886	0.168544491	24.76931674	1.92E-135	9.49E-133	112.8440221	1.892722087	0.171689003	11.02413116	2.92E-28	3.65E-26	106.2010835	1.127041485	0.179755563	6.26985595	3.61E-10	2.85E-09	411.6443598	4.944674683	0.23815089	20.76278063	9.40E-96	4.37E-92
24	HJURP	719.479066	1.920025316	0.21699738	8.848149746	8.90E-19	2.65E-17	125.6267058	3.10462309	0.174125279	17.8298241	4.15E-71	1.73E-69	336.2240434	3.611575076	0.208470587	17.32414689	3.09E-67	4.63E-65	986.9111254	4.849717342	0.19179164	25.28638544	4.51E-141	2.97E-138	167.3633501	2.465924065	0.185615718	13.28510374	2.82E-40	1.73E-37	95.65780554	1.327508841	0.222636861	5.962664193	2.48E-09	1.80E-08	530.2382624	5.632015618	0.272291007	20.68381062	4.85E-95	1.97E-91
25	IQGAP3	1220.655172	1.627517527	0.217976879	7.466468607	8.24E-14	1.29E-12	310.322723	2.590547427	0.164712644	15.72767795	9.77E-56	2.37E-54	713.4245592	3.498628173	0.192704423	18.15541187	1.16E-73	2.45E-71	1466.589177	3.965035503	0.167403922	23.68543971	5.09E-124	1.46E-121	165.0412405	1.758743967	0.178491522	9.853375399	6.63E-23	3.62E-21	154.6489464	2.453226556	0.194037479	12.64305521	1.22E-36	8.49E-35	755.1102492	5.191351863	0.261575547	19.84647236	1.18E-87	1.75E-84
26	KIF14	701.2996099	2.343251458	0.235718217	9.940901007	2.76E-23	1.39E-21	82.83553805	2.913032985	0.166124759	17.53521268	7.72E-69	2.96E-67	244.9629437	3.646622676	0.21567099	16.90826695	3.91E-64	4.72E-62	663.545821	4.761510488	0.187294408	25.42259826	1.42E-142	1.02E-139	54.49545465	1.679138552	0.192412738	8.726753585	2.62E-18	7.23E-17	48.68346188	1.005924838	0.208733277	4.819187693	1.44E-06	7.60E-06	215.4282443	4.850764064	0.272104736	17.82682703	4.38E-71	2.34E-68
27	KIF18B	796.7678834	1.794966634	0.226693138	7.918045729	2.41E-15	4.60E-14	88.30527288	3.514705631	0.191170725	18.38516661	1.73E-75	8.28E-74	269.2800015	3.557131302	0.214474954	16.58529926	8.90E-62	9.78E-60	1018.589695	5.031353958	0.202144177	24.88992773	9.56E-137	5.18E-134	64.23574446	1.690989837	0.198657401	8.512090828	1.71E-17	4.20E-16	83.04078264	1.59201757	0.216669772	7.347668084	2.02E-13	2.18E-12	429.972905	5.370798445	0.288696627	18.6036065	3.00E-77	2.13E-74
28	KIF20A	1491.450604	1.502605009	0.250172697	6.006270969	1.90E-09	1.61E-08	300.5499651	2.7273507	0.157564599	17.30941284	3.99E-67	1.44E-65	473.7038385	3.265319839	0.175990572	18.5539475	7.58E-77	1.85E-74	1350.650615	4.078547717	0.182124755	22.39425235	4.48E-111	8.63E-109	179.7576544	1.755740092	0.17406877	10.08647381	6.34E-24	4.00E-22	155.8324479	1.45704791	0.178619692	8.157263611	3.43E-16	4.74E-15	696.027437	5.083231615	0.28541671	17.8098599	5.93E-71	3.11E-68
29	KIF4A	1160.794713	1.886456052	0.215886654	8.738178176	2.37E-18	6.68E-17	197.8018134	2.599407998	0.138090201	18.82398598	4.80E-79	2.64E-77	488.0552591	3.871048656	0.211272843	18.32250945	5.47E-75	1.23E-72	1243.272018	4.633588751	0.178821017	25.91188009	4.89E-148	4.61E-145	175.9211259	2.005907877	0.18634218	10.7646475	5.06E-27	5.21E-25	114.7822187	1.402794883	0.176169284	7.962766543	1.68E-15	2.19E-14	534.4069812	4.359940502	0.341629882	12.76217547	2.67E-37	2.43E-35
30	KISS1R	7.633884097	2.048181334	0.37806367	5.417556613	6.04E-08	4.09E-07	231.0690332	6.836326062	0.338782922	20.1790752	1.50E-90	1.15E-88	34.08643547	4.830825822	0.370826621	13.02718183	8.57E-39	2.85E-37	18.92673856	3.43748207	0.330510576	10.40052064	2.47E-25	2.21E-24	2.395872875	1.854635858	0.412281007	4.498475133	6.84E-06	3.11E-05	21.53787921	3.848458188	0.281861016	13.6537441	1.92E-42	1.99E-40	166.6400096	4.211033966	0.567749937	7.417057572	1.20E-13	1.68E-12
31	LHX2	64.88856159	4.019391719	0.384049202	10.46582495	1.24E-25	8.02E-24	7.515258266	2.736904275	0.390127146	7.015416131	2.29E-12	8.11E-12	13.18662905	3.77010795	0.397108852	9.493890481	2.23E-21	2.50E-20	117.1114323	5.971760915	0.386565356	15.44825688	7.75E-54	2.35E-52	4.846545197	1.175613535	0.315674042	3.724137492	0.000195984	0.000684614	116.5371629	1.395642483	0.383770858	3.636655717	0.000276201	0.001067516	14.26779102	4.445994603	0.624557822	7.118627683	1.09E-12	1.36E-11
32	MCM10	568.8193254	1.43713052	0.23219939	6.189208843	6.05E-10	5.53E-09	74.87156148	2.987367192	0.152889218	19.53942357	5.08E-85	3.27E-83	235.9431062	3.380375674	0.212714996	15.89157197	7.25E-57	6.10E-55	692.5723677	4.502487906	0.194005922	23.20799213	3.78E-119	9.34E-117	57.38297018	1.163191606	0.185605546	6.26700888	3.68E-10	3.25E-09	67.09033659	1.303345404	0.164882548	7.90468984	2.69E-15	3.44E-14	267.2906061	5.260280604	0.279554977	18.81662299	5.52E-79	4.28E-76
33	MELK	1173.80399	1.695644725	0.248154432	6.833022127	8.31E-12	9.95E-11	141.4496117	3.043353026	0.166773056	18.24846949	2.13E-74	9.83E-73	389.614529	3.524174603	0.207821998	16.95765917	1.69E-64	2.11E-62	1185.168012	4.724776427	0.198021904	23.8598677	8.00E-126	2.49E-123	120.8144085	1.931796294	0.184386779	10.47686992	1.10E-25	9.33E-24	94.111653	1.832366335	0.208876868	8.772471341	1.75E-18	2.92E-17	444.4761788	5.360382609	0.330755164	16.2064971	4.54E-59	1.23E-56
34	MKI67	7487.747075	1.6169958	0.237971638	6.794909744	1.08E-11	1.27E-10	945.6517779	3.18035399	0.169060164	18.81196565	6.03E-79	3.30E-77	1978.499762	3.189964688	0.206326729	15.46074374	6.39E-54	4.61E-52	5679.856367	4.164773527	0.182128929	22.86717186	9.86E-116	2.22E-113	875.7822095	2.153636864	0.191567279	11.24219584	2.53E-29	3.90E-27	657.3404886	1.357214146	0.218053181	6.224234571	4.84E-10	3.77E-09	2318.777038	5.368460916	0.32175676	16.68484265	1.69E-62	5.73E-60
35	MMP9	4509.467316	4.298473084	0.299653381	14.34481755	1.15E-46	6.67E-44	729.8883154	3.178950233	0.241106622	13.18483172	1.07E-39	1.34E-38	1761.290471	2.695050008	0.246018715	10.95465442	6.31E-28	1.12E-26	5249.885079	2.576669933	0.238974557	10.78219355	4.18E-27	4.10E-26	294.0024472	1.682669108	0.246181121	6.835085882	8.20E-12	9.10E-11	1779.979195	1.175101513	0.307060017	3.826944078	0.000129744	0.00052646	3686.625625	3.357688748	0.437071086	7.682248625	1.56E-14	2.43E-13
36	MRPL40P1	3.586991082	1.398043933	0.406976203	3.435198231	0.000592121	0.001898412	2.280499076	1.366624694	0.255036591	5.358543601	8.39E-08	2.18E-07	3.077886876	1.725461328	0.366409134	4.709111116	2.49E-06	8.12E-06	3.969277491	1.527250837	0.278306812	5.487651655	4.07E-08	1.31E-07	1.37773643	1.25063654	0.413235783	3.026447834	0.002474455	0.006838966	5.725145144	2.53437782	0.343967077	7.368082554	1.73E-13	1.88E-12	2.965861834	2.6932549	0.654167522	4.117072175	3.84E-05	0.00015588
37	MYBL2	3146.626237	2.135949476	0.222383039	9.604821886	7.63E-22	3.30E-20	238.8121596	3.76543331	0.211336186	17.81726726	5.19E-71	2.15E-69	1220.592618	4.038664135	0.213913318	18.87990977	1.67E-79	4.50E-77	3408.876881	5.023889523	0.19877541	25.27420033	6.14E-141	3.98E-138	285.9746533	2.203979867	0.182313922	12.08892796	1.21E-33	3.34E-31	422.5306548	1.211299991	0.232786232	5.203486384	1.96E-07	1.14E-06	1618.544882	6.254880778	0.265991459	23.51534445	2.84E-122	3.08E-118
38	NCAPH	1002.088746	1.594446937	0.199603607	7.988066772	1.37E-15	2.69E-14	135.3920737	2.225807646	0.127149203	17.50547853	1.30E-68	4.95E-67	354.7511065	2.871171238	0.176504326	16.26686045	1.70E-59	1.65E-57	1263.234543	4.220284972	0.151258789	27.9010893	2.59E-171	1.71E-167	121.1469971	1.336201164	0.150440271	8.881938048	6.57E-19	1.95E-17	130.6191324	1.209159119	0.185149096	6.530731959	6.54E-11	5.58E-10	456.7798842	4.604841281	0.26090261	17.64965584	1.02E-69	5.21E-67
39	NEIL3	193.3036801	1.578987941	0.264720317	5.964740293	2.45E-09	2.05E-08	44.93198859	3.433112515	0.227617319	15.0828264	2.10E-51	4.30E-50	94.15879082	3.641323956	0.222244012	16.38435127	2.47E-60	2.50E-58	186.872478	4.189291628	0.202263163	20.71208399	2.70E-95	3.33E-93	20.44997294	2.084115399	0.219629252	9.48924324	2.33E-21	9.90E-20	21.34629422	1.17352888	0.23499021	4.993947964	5.92E-07	3.27E-06	72.54600059	4.854292255	0.350888644	13.83428144	1.58E-43	2.05E-41
40	NEK2	692.0636893	1.690703904	0.236299528	7.154918662	8.37E-13	1.14E-11	100.4761639	2.580394272	0.155067857	16.64041998	3.55E-62	1.07E-60	363.7316497	3.539091947	0.220317316	16.06361227	4.59E-58	4.12E-56	868.8388005	4.712258871	0.179755966	26.21475649	1.80E-151	2.04E-148	94.10569207	1.812895361	0.182269629	9.9462284	2.62E-23	1.50E-21	66.72539059	1.18140277	0.20723626	5.700753201	1.19E-08	8.01E-08	419.6337525	4.835435388	0.359585003	13.44726657	3.19E-41	3.73E-39
41	NFE2L3	598.7743488	1.100005937	0.209097897	5.260722141	1.43E-07	9.12E-07	1209.727404	1.587156807	0.117683462	13.48665971	1.87E-41	2.53E-40	845.9790426	1.369274596	0.152290754	8.991186678	2.45E-19	2.39E-18	1447.19455	1.222940812	0.154546519	7.913091951	2.51E-15	1.32E-14	333.4383327	1.124670378	0.14633954	7.685348612	1.53E-14	2.48E-13	1183.16291	2.634727675	0.235933515	11.16724633	5.90E-29	2.27E-27	500.1282764	1.750863037	0.320392806	5.464738925	4.64E-08	3.09E-07
42	PIMREG	348.6853197	1.501120629	0.21120373	7.107453205	1.18E-12	1.58E-11	81.0369535	2.739303228	0.163768885	16.72664024	8.38E-63	2.59E-61	145.2154447	3.188725168	0.202056039	15.78139007	4.18E-56	3.38E-54	450.7766553	4.232195805	0.182902938	23.13902582	1.88E-118	4.55E-116	73.19126614	1.71306799	0.168255313	10.18136045	2.40E-24	1.62E-22	43.81937127	1.65405973	0.160789134	10.28713626	8.05E-25	2.26E-23	270.5475924	4.564386146	0.260000487	17.55529848	5.42E-69	2.56E-66
43	PKMYT1	1070.375849	1.839252766	0.228662203	8.043536448	8.73E-16	1.76E-14	69.4988493	2.982573028	0.178740995	16.68656383	1.64E-62	5.01E-61	218.039339	2.90990894	0.188630503	15.42650261	1.09E-53	7.76E-52	758.5463242	4.231553496	0.180204827	23.48190976	6.24E-122	1.74E-119	119.9471549	1.218353965	0.178868252	6.811460128	9.66E-12	1.06E-10	100.9487494	1.89680921	0.176284352	10.75994089	5.32E-27	1.78E-25	400.38359	5.196095242	0.328659947	15.80994367	2.66E-56	6.05E-54
44	RAET1K	9.9916	3.611145	0.3338	10.815	2.90E-	2.22E-	8.3852	1.325002	0.1724	7.6824	1.56E-	6.29E-	6.9241	2.454617	0.2926	8.3865	5.01E-	4.11E-	23.618	2.804111	0.2427	11.549	7.40E-	8.76E-	8.5921	1.388467	0.2354	5.8975	3.69E-	2.81E-	6.6637	1.045697	0.2215	4.7206	2.35E-	1.21E-	14.177	4.847319	0.5017	9.6598	4.46E-	1.49E-

		31412	646	79898	70249	27	25	73755	22	71048	61683	14	14	11389	225	85998	20847	17	16	30285	096	84204	80864	31	30	25629	097	30319	71326	09	08	63542	603	14166	80488	06	05	84458	655	99717	69256	22	20
45	RDM1	20.40503845	1.593075741	0.252044468	6.320613791	2.61E-10	2.51E-09	14.06353456	1.969482936	0.153064094	12.86704732	6.90E-38	8.07E-37	17.0432873	3.231759934	0.264517014	12.21758815	2.50E-34	6.64E-33	36.72114921	4.150752079	0.256865729	16.15922879	9.78E-59	3.58E-57	9.409025312	1.260522969	0.251308642	5.015836138	5.28E-07	2.89E-06	15.71972785	1.942788295	0.202364772	9.600427369	7.96E-22	1.76E-20	22.11642715	3.525932323	0.430280126	8.194504247	2.52E-16	4.70E-15
46	RRM2	3994.829813	1.253795749	0.233765904	5.363467158	8.16E-08	5.40E-07	557.9639495	2.656941593	0.156724173	16.95297884	1.83E-64	6.00E-63	1361.395599	3.240785348	0.183680531	17.64359743	1.14E-69	2.00E-67	3631.940149	4.129002962	0.192982275	21.39576273	1.46E-101	2.17E-99	424.7164303	1.821982457	0.17245443	10.56500815	4.33E-26	3.89E-24	403.2651241	1.533655604	0.228922812	6.699444184	2.09E-11	1.87E-10	1007.091687	4.680122874	0.299390221	15.6321835	4.39E-55	9.42E-53
47	SKA3	537.5521464	1.136709545	0.231739481	4.905118197	9.34E-07	5.14E-06	91.69989972	2.385182437	0.128513896	18.559724	6.81E-77	3.42E-75	152.9092105	3.091272667	0.192095369	16.09238514	2.89E-58	2.62E-56	429.2275189	4.15529083	0.171265569	24.26226625	4.91E-130	1.83E-127	90.38995587	1.831780219	0.159460867	11.48733384	1.53E-30	2.86E-28	54.42686098	1.211557655	0.170459915	7.107580999	1.18E-12	1.19E-11	264.7640496	4.575408635	0.254563092	17.97357426	3.14E-72	1.82E-69
48	SPC24	497.3122778	1.341067974	0.190688145	7.032781043	2.02E-12	2.63E-11	132.5760174	2.142537487	0.13522807	15.84388124	1.55E-56	3.88E-55	181.1334548	2.577362983	0.159350656	16.17415982	7.67E-59	7.21E-57	564.8992224	3.579396224	0.145418756	24.61440546	8.86E-134	3.89E-131	120.6548213	1.255651314	0.131661014	9.537001711	1.47E-21	6.44E-20	134.0721013	1.806352172	0.16000822	11.28912107	1.49E-29	6.06E-28	526.997415	4.309742761	0.268255365	16.06582133	4.43E-58	1.13E-55
49	TMEM132A	4774.970573	1.846273126	0.16760377	11.01570163	3.21E-28	2.73E-26	1636.7883	1.078993589	0.141883003	7.604812165	2.85E-14	1.13E-13	1419.205626	1.969845957	0.168808598	11.66910913	1.83E-31	4.09E-30	4468.068003	3.061727899	0.13262091	23.0863135	6.35E-118	1.53E-115	1949.526578	1.716737955	0.132658204	12.94106133	2.64E-38	1.31E-35	548.277857	1.43457975	0.119973664	11.95745549	5.94E-33	3.07E-31	4792.466996	3.56061929	0.195824166	18.18273687	7.07E-74	4.34E-71
50	TOMM40P2	9.284477534	2.175619944	0.328916557	6.614504184	3.73E-11	4.07E-10	7.115346224	2.703806383	0.240205448	11.2562242	2.16E-29	1.78E-28	6.283454815	1.562574223	0.256012838	6.10349947	1.04E-09	4.66E-09	12.55329704	2.134585475	0.235241132	9.074031646	1.15E-19	7.66E-19	5.729472699	1.146408614	0.24886005	4.606639811	4.09E-06	1.93E-05	2.978840543	1.428041719	0.254903208	5.60229011	2.12E-08	1.38E-07	8.504145736	1.508922429	0.467779275	3.225714584	0.001256586	0.003745832
51	TOP2A	5783.210009	1.523489973	0.22884155	6.657401056	2.79E-11	3.09E-10	968.8804038	2.960463383	0.150545239	19.66494196	4.31E-86	2.85E-84	3006.433956	3.786274125	0.201979151	18.74586612	2.09E-78	5.40E-76	8286.035954	4.831857678	0.196760306	24.55707547	3.63E-133	1.54E-130	893.1429569	1.644327057	0.180458754	9.11192733	8.09E-20	2.74E-18	757.0627998	1.53662236	0.191821397	8.010693188	1.14E-15	1.51E-14	2594.411849	4.941539678	0.281639641	17.54561134	6.43E-69	2.99E-66
52	TPX2	4235.438137	1.927838585	0.224478108	8.588091745	8.84E-18	2.34E-16	573.8160955	2.769436477	0.146769874	18.86924344	2.04E-79	1.14E-77	1383.21823	3.56818683	0.203212959	17.55885478	5.09E-69	8.39E-67	4934.793392	4.979741222	0.183337628	27.16158858	1.85E-162	4.87E-159	426.4337565	1.662325859	0.170463858	9.751778928	1.81E-22	9.21E-21	367.1485262	1.427784159	0.192493329	7.417317593	1.20E-13	1.32E-12	1504.371797	4.899101975	0.242161295	20.23073905	5.25E-91	1.22E-87
53	TROAP	629.4682036	1.568856388	0.222134425	7.062644107	1.63E-12	2.15E-11	81.82419837	2.989369699	0.174120415	17.1684044	4.58E-66	1.59E-64	229.9637113	3.238908683	0.210610395	15.3786744	2.28E-53	1.61E-51	843.3911694	4.750749151	0.177902243	26.7042678	4.20E-157	7.22E-154	95.77560934	1.565156011	0.162397418	9.637813383	5.54E-22	2.63E-20	89.22625709	1.066379769	0.17133697	6.223874332	4.85E-10	3.78E-09	606.7617007	5.499196419	0.270280977	20.34622074	5.01E-92	1.36E-88
54	UBE2C	1806.114381	1.807551271	0.223450117	8.089283152	6.00E-16	1.24E-14	252.9133962	3.090841572	0.177146977	17.44789338	3.57E-68	1.33E-66	786.6030446	3.884826638	0.197406487	19.67932613	3.24E-86	1.30E-83	2088.059564	4.916432995	0.189050304	26.00595121	4.24E-149	4.30E-146	315.8672746	1.840722588	0.19388669	9.493805825	2.23E-21	9.51E-20	204.3169417	1.739001122	0.233409324	7.450435552	9.30E-14	1.04E-12	1232.882191	5.594168484	0.25671066	21.79172644	2.78E-105	2.26E-101
55	UHRF1	1115.701176	1.667411296	0.21468981	7.766606612	8.06E-15	1.44E-13	165.3791855	3.209117242	0.173751596	18.46956989	3.63E-76	1.78E-74	376.6460082	3.443618894	0.204101658	16.87207699	7.22E-64	8.69E-62	1022.934617	4.229147901	0.172517995	24.51424215	1.04E-132	4.29E-130	150.1186493	1.168149171	0.178082023	6.559613097	5.39E-11	5.37E-10	154.1554339	2.196679061	0.206139648	10.65626666	1.63E-26	5.23E-25	535.1568777	4.777101043	0.318593442	14.99434834	7.99E-51	1.42E-48

Supplementary Table 2. Markers for cell identities, and pecentages of various cells in tumor microenviroments

**Cell types**	**Markers**	**Clusters**	**Cell numbers**	**Percentages (%)**
Melanoma cells	DCT, PMEL	1, 4, 5, 11, 24, 26, 31, 34	7217	24.75
Melanocytes	PMEL	7, 22, 27	1919	6.58
Keratinocytes	KRT10, PMEL	2, 9, 13, 14, 17, 23, 25	6506	22.31
Hair follicles	KRT5, KRT10	36	45	1.54
Endothelial cells	VWF, PECAM1	16	715	2.45
Adipocytes	APOD	10	1081	3.71
Fibroblasts	COL3A1, COL1A2, COL1A1	19, 21, 32, 35	1216	4.17
Monocytes	CD68, LYZ	29	226	0.78
γδ T cells	NKG7, CD3D,	20	478	1.64
Treg	FOXP3, CD4, CD3D, CD3E, CD3G	28	227	0.78
Exhausted CD4+ T cells	CTLA4, PDCD1,BTLA, CD4, CD3D, CD3E, CD3G	12	1000	3.43
Exhausted CD8+ T cells	CTLA4, PDCD1,BTLA, CD8, CD3D, CD3E, CD3G	15, 18, 33	1432	4.91
Cytotoxic T lymphocytes	GZMB,CD8A, NKG7, KLRD1, CD3D, CD3E, CD3G	8	1159	3.97
T memory cells	CCR7, CD3D, CD3E, CD3G, NKG7	0	2401	8.23
B cells	CD79A, MS4A1	6	1274	4.37
NK cells	KLRD1, NKG7 (CD3Dlow, CD3Elow, CD3Glow)	3, 30	2262	7.76

Supplementary Table 3. PCEs with the system method

Materials	Laser condition	Time constant method	Integrating sphere method	ΔT_{max} (Increased temperature)	System method
Graphene (1 g/L)	808 nm, 600 s, 200 mW	~70%	~65%	~15.2℃	43.5%
Graphene oxide (1 g/L)		~63%	~60%	~3.8℃	10.2%
Gold nanorod (0.15 g/L)	650 nm, 600 s, 200 mW	~52%	~56%	~4.9℃	17.0%

Supplementary Materials 1

Photothermal therapy: A theory study of photothermal conversion efficiency (PCE) and photoacoustic imaging

Yujuan Zhang [1*]

[1] Department of Otolaryngology, The First Dongguan Affiliated Hospital, and Nursing College, Guangdong Medical University, Dongguan, 523808, China

* Correspondence should be addressed to Dr Yujuan Zhang (zhangyj@gdmu.edu.cn).

Supplementary Note 1: Introduction of absorption coefficient

Photothermal therapy is a non-invasive tumor therapy via photothermal agent converting light into thermal energy to kill tumor cells. The wavelength of light for photothermal therapy is mainly located at near infrared (NIR) including such as the first therapy window (650-950 nm) and the second therapy window (1,000-1,350 nm) which possess highly biological safety. For biomacromolecules, the absorption coefficient (α') of the relevant electromagnetic waves at certain concentration is shown in Supplementary Fig. 6[1]. It can also be obtained from lambert-beer law:

$$A^{'} = \lg(1/T^{'}) = \alpha^{'} b \tag{1}$$

Where A' is absorbance, T' is transmission ratio, b is the measure thickness (cm). Due to the complex tumor microenvironment (A large number of tumor neovascularization, tumor cells, immunocytes), when the absorption of melanin is not considered, the electromagnetic wave length of about 650-800 nm may have better biological tissue penetration as shown in the pink filled area of Supplementary Fig. 6; the melanin content in the skin varies greatly due to different skin colors, and the absorption of electromagnetic waves by melanin in the skin can be effectively avoided when the tissue is locally irradiated). Literature shows that the near-infrared band can penetrate several centimeters into the tissue[2].

Supplementary Note 2: Photothermal conversion efficiency (PCE)

The selection of photothermal agent generally possesses the following characteristics: 1) high photothermal conversion

efficiency (PCE); 2) easy preparation; 3) good bio-compatibility and quick elimination. The photothermal agents, such as noble metal nanoparticles, carbon nanomaterals, metal and non-metal quantum dots, and organic dyes[3], in addition, composite materials and new materials[4], normally possess high PCE and easy preparation. But particle size and surface characterization mainly affect the biocompatibility, in vivo distribution and elimination[5]. Here, the following research method is proposed in this study (96 well plate is used to record the temperature change curve under laser irradiation, as shown in Supplementary Fig. 7). As follows (Supplementary Fig. 7), according to the first law of thermodynamics (Law of conservation of energy):

$t_0 \rightarrow t_2$
$$E_{in} = \xi E_{laser,\,in} = E_{out} \tag{2}$$

where ξ is PCE (Photothermal Conversion Efficiency), $E_{laser,\,in}$ is the incident laser energy. When only heat conduction is considered for energy transfer, the heat conduction formula of one-dimensional single-layer thin-walled (A>>d) with uniform mass is as follows:

$$q = {}^{\Delta T}/_{R};\ R = {}^{d}/_{(\lambda A)} \tag{3}$$

where q is heat conduction velocity (W or J/s), ΔT is temperature difference on both sides of thin wall, i.e. heat conduction driving force (°C), R is the thermal resistance of heat conduction, d is the thin wall thickness (m), A is the area of thin wall (m^2), λ is the thermal conductivity (W/(m·°C)), the difference is small within a certain temperature change, which is close to a constant, such as transparent quartz glass λ is 0.00331 W/（m·°C） at 20 °C and 0.00367 W/ (m·°C) at 100 °C (the killing temperature of general tumor is between 43-46 °C, and the basic body temperature of human body is ~ 37 °C).

$$E_{laser,\,in} = ISt_1 \tag{4}$$

where I is the light intensity (W/cm^2), S is the light spot area (cm^2) irradiated to the sample, and t_1 is the irradiation time (s).

$$\xi = PCE = \frac{E_{in}}{E_{laser,\,in}} = \frac{E_{out}}{E_{laser,\,in}} = \frac{1}{ISRt_1}\int_{t_0}^{t_2} \Delta T\, dt = \frac{\lambda A}{ISdt_1}\int_{t_0}^{t_2} \Delta T\, dt \tag{5}$$

The PCE of the material in Supplementary Fig. 7 can be expressed as:

$$\xi_{materials} = \frac{\lambda A}{ISdt_1}\left[\int_{t_0}^{t_1}\left(\Delta T_{(materials+medium)1} - \Delta T_{medium1}\right)dt + \int_{t_1}^{t_2}\left(\Delta T_{(materials+medium)2} - \Delta T_{medium2}\right)dt\right] \tag{6}$$

At the stage of laser on,

$$(m_{materials}C_{materials} + m_{medium}C_{medium})d\Delta T_{(materials+medium)1} = \xi ISdt - \frac{\lambda A}{d}\Delta T_{(materials+medium)1}dt \tag{7}$$

$$\Delta T_{(materials+medium)1} = -\frac{\xi ISd}{\lambda A} e^{-\frac{\lambda A}{(m_{materials}C_{materials}+m_{medium}C_{medium})d}t} + \frac{\xi ISd}{\lambda A} \tag{8}$$

At the stage of laser off,

$$-(m_{materials}C_{materials} + m_{medium}C_{medium})d\Delta T_{(materials+medium)2} = \frac{\lambda A}{d}\Delta T_{(materials+medium)2}dt \tag{9}$$

$$\Delta T_{(materials+medium)2} = \frac{\xi ISd\left(e^{\frac{\lambda A}{(m_{materials}C_{materials}+m_{medium}C_{medium})d}t_1} - 1\right)}{\lambda A} e^{-\frac{\lambda A}{(m_{materials}C_{materials}+m_{medium}C_{medium})d}t} \tag{10}$$

Therefore,

$$\Delta T_{max} = \frac{\xi \mathrm{ISd}}{\lambda \mathrm{A}} \quad (11)$$

where m is the mass (kg), C is the specific heat capacity (J / (kg·°C)). It can be seen from the above equation (11) that ΔT_{max} is proporational to ξI. Therefore, ξ can be quickly obtained according to ΔT_{max}. As a fast and simple method for measuring and comparing the PCEs, the data in Supplementary Table 3 (Data from the reference[3]) are recalculated according to the equation (5), which is listed in the last column "System method":

Through this system method, it can be clearly found that the PCE of graphene (1 g/L) is higher than that of graphene oxide (1 g/L), with a ratio of 4.26. According to equation (11), the ΔT_{max} ratio and the PCE ratio are the same under the irradiation condition. The actual measured ΔT_{max} ratio is 15.2:3.8 = 4, which is consistent with 4.26 by this system method. Therefore, this system method can quickly and simply measure the PCE and exhibit the PCE of the mixture of photothermal agent and medium.

Supplementary Note 3: Photoacoustic imaging

Photoacoustic imaging is a method that uses photothermal agents to convert light energy into thermal energy and generates a temperature rise, and thermoelastic expansion and pressure take place, resulting in the emission of acoustic waves for imaging, including photothermal microscopy (PAM) and photothermal computer tomography (PACT). Some natural photothermal agents exist in the body, such as oxyhemoglobin, hypoxic hemoglobin, melanin, etc. (Supplementary Fig. 6). According to the equation of state of liquids and solids:

$$\mathrm{dv} = \left(\frac{\partial \mathrm{V}}{\partial \mathrm{p}}\right)_{\mathrm{T}} \mathrm{dp} + \left(\frac{\partial \mathrm{V}}{\partial \mathrm{T}}\right)_{\mathrm{p}} \mathrm{dT} \quad (12)$$

, it can be concluded that:

$$\frac{dV}{V} =- \kappa dp + \alpha dT \quad (13)$$

where κ is the isothermal compressibility, α is the thermal coefficient of volume expansion, and dp and dT are changes in pressure and temperature. As shown in reference[6], when in a short time (pulse laser, ns), let $\frac{dV}{V} = 0$, the equation (12) can be rewritten to:

$$\mathrm{dp} = \frac{\alpha \mathrm{dT}}{\kappa} \quad (14)$$

According to equation (8) at the stage of laser on:

$$\frac{d\Delta T_{(material+medium)1}}{dt} = \frac{\xi IS}{m_{materials}c_{materials}+m_{medium}c_{medium}} e^{-\frac{\lambda A}{(m_{materials}c_{materials}+m_{medium}c_{medium})d}t} \tag{15}$$

According to equation (10) at the stage of laser off:

$$\frac{d\Delta T_{(material+medium)2}}{dt} = \frac{\xi IS}{m_{materials}c_{materials}+m_{medium}c_{medium}} \left[1 - e^{\frac{\lambda A}{(m_{materials}c_{materials}+m_{medium}c_{medium})d}t_1}\right] e^{-\frac{\lambda A}{(m_{materials}c_{materials}+m_{medium}c_{medium})d}t} \tag{16}$$

According to equation (14),

$$\frac{dp}{dt} \propto \frac{d\Delta T}{dt} \tag{17}$$

Therefore, according to the temperature change curve of photothermal agent compound as shown in Supplementary Fig. 7, the photoacoustic pressure of $\frac{d\Delta T}{dt}$ and $\frac{dp}{dt}$ can be obtained via equations of (15) and (16).

In conclusion, photothermal effects depend on a combination of factors. Ultimately, the appropriate choice of photothermal agent mainly depend on PCEs. In this study, we determined the PCE and photoacoustic pressure for photothermal agent suspension system for simulating being dispersing in vivo.

Acknowledgments

Acknowledgments for Gao Xiao's supporting.

Conflict of interest statement

All authors declare nothing to disclose regarding conflict of interest to this manuscript.

Availability of Data and Materials

All data generated or analyzed during this study are included in this published article.

Supplementary References

1. Beard, P. Biomedical photoacoustic imaging. *Interface Focus* **1**, 602-631 (2011).
2. Weissleder, R. A clearer vision for in vivo imaging. *Nat Biotechnol* **19**, 316-317 (2001).
3. Savchuk, O.S., Carvajal, J.J., Massons, J., Aguiló, M. & Díaz, F. Determination of photothermal conversion efficiency of graphene and graphene oxide through an integrating sphere method. *Carbon* **103**, 134-141 (2016).

4. Hu, J.J., Cheng, Y.J. & Zhang, X.Z. Recent advances in nanomaterials for enhanced photothermal therapy of tumors. *Nanoscale* **10**, 22657-22672 (2018).
5. Müller, R.H., Lück, M., Harnisch, S. & Thode, K. Intravenously injected particles surface properties and interaction with blood proteins-the key determining organ distribution. (1997).
6. Xia, J., Yao, J. & Wang, L.V. Photoacoustic tomography: principles and advances. *Electromagn Waves (Camb)* **147**, 1-22 (2014).
