# Supplementary material for: Tumor-targeted nanodrug FSGG/siGal-9 for transdermal photothermal immunotherapy of melanoma
Source: Commun Biol. 2024 Feb 16;7:188. doi: 10.1038/s42003-024-05891-6 (PMC10873409; doi:10.1038/s42003-024-05891-6)
Supplement: Supplementary file 3 — Description of Additional Supplementary Files [file 42003_2024_5891_MOESM3_ESM.pdf]

Description of Additional Supplementary Files

File name: Supplementary Data

Description: Proteomics of exosome or exosome/siGal-9 and the numerical source data for all figures.
