# Supplementary material for: Tumor-targeted nanodrug FSGG/siGal-9 for transdermal photothermal immunotherapy of melanoma
Source: Commun Biol. 2024 Feb 16;7:188. doi: 10.1038/s42003-024-05891-6 (PMC10873409; doi:10.1038/s42003-024-05891-6)
Supplement: Supplementary file 5 — Reporting Summary [file 42003_2024_5891_MOESM5_ESM.pdf]

nature portfolio

Corresponding author(s): DAPR COMMSBIO-23-0815C

Last updated by author(s): Jan 2, 2024

Reporting Summary

Nature Portfolio wishes to improve the reproducibility of the work that we publish. This form provides structure for consistency and transparency in reporting. For further information on Nature Portfolio policies, see our Editorial Policies and the Editorial Policy Checklist.

Statistics

For all statistical analyses, confirm that the following items are present in the figure legend, table legend, main text, or Methods section.

n/a	Confirmed	
☐	☒	The exact sample size (*n*) for each experimental group/condition, given as a discrete number and unit of measurement
☐	☒	A statement on whether measurements were taken from distinct samples or whether the same sample was measured repeatedly
☐	☒	The statistical test(s) used AND whether they are one- or two-sided<br>*Only common tests should be described solely by name; describe more complex techniques in the Methods section.*
☒	☐	A description of all covariates tested
☒	☐	A description of any assumptions or corrections, such as tests of normality and adjustment for multiple comparisons
☐	☒	A full description of the statistical parameters including central tendency (e.g. means) or other basic estimates (e.g. regression coefficient) AND variation (e.g. standard deviation) or associated estimates of uncertainty (e.g. confidence intervals)
☐	☒	For null hypothesis testing, the test statistic (e.g. *F*, *t*, *r*) with confidence intervals, effect sizes, degrees of freedom and *P* value noted<br>*Give P values as exact values whenever suitable.*
☒	☐	For Bayesian analysis, information on the choice of priors and Markov chain Monte Carlo settings
☒	☐	For hierarchical and complex designs, identification of the appropriate level for tests and full reporting of outcomes
☒	☐	Estimates of effect sizes (e.g. Cohen's *d*, Pearson's *r*), indicating how they were calculated

Our web collection on statistics for biologists contains articles on many of the points above.

Software and code

Policy information about availability of computer code

Data collection	The Cancer Genome Atlas database; The GEO database
Data analysis	R 4.3.0; R studio

For manuscripts utilizing custom algorithms or software that are central to the research but not yet described in published literature, software must be made available to editors and reviewers. We strongly encourage code deposition in a community repository (e.g. GitHub). See the Nature Portfolio guidelines for submitting code & software for further information.

Data

Policy information about availability of data

All manuscripts must include a data availability statement. This statement should provide the following information, where applicable:

- Accession codes, unique identifiers, or web links for publicly available datasets
- A description of any restrictions on data availability
- For clinical datasets or third party data, please ensure that the statement adheres to our policy

The Cancer Genome Atlas database; The GEO database (GSE215120); http://www.ncbi.nlm.nih.gov/gene/; Supplementary Data

Research involving human participants, their data, or biological material

Policy information about studies with human participants or human data. See also policy information about sex, gender (identity/presentation), and sexual orientation and race, ethnicity and racism.

Reporting on sex and gender	No
Reporting on race, ethnicity, or other socially relevant groupings	No
Population characteristics	No
Recruitment	No
Ethics oversight	No

Note that full information on the approval of the study protocol must also be provided in the manuscript.

Field-specific reporting

Please select the one below that is the best fit for your research. If you are not sure, read the appropriate sections before making your selection.

☒ Life sciences ☐ Behavioural & social sciences ☐ Ecological, evolutionary & environmental sciences

For a reference copy of the document with all sections, see nature.com/documents/nr-reporting-summary-flat.pdf

Life sciences study design

All studies must disclose on these points even when the disclosure is negative.

Sample size	Data were obtained from publicly available datasets.
Data exclusions	No
Replication	Yes
Randomization	Yes
Blinding	Yes

Reporting for specific materials, systems and methods

We require information from authors about some types of materials, experimental systems and methods used in many studies. Here, indicate whether each material, system or method listed is relevant to your study. If you are not sure if a list item applies to your research, read the appropriate section before selecting a response.

Materials & experimental systems

n/a	Involved in the study	
☐	☒	Antibodies
☐	☒	Eukaryotic cell lines
☒	☐	Palaeontology and archaeology
☐	☒	Animals and other organisms
☒	☐	Clinical data
☒	☐	Dual use research of concern
☒	☐	Plants

Methods

n/a	Involved in the study	
☐	☒	ChIP-seq
☐	☒	Flow cytometry
☒	☐	MRI-based neuroimaging

Antibodies

Antibodies used	FITC-ANTI-CD4 (#11-0041-81, Thermofisher), PE-Cyanine5-ANTI-CD4 (#15-0041-81, Thermofisher), PE-ANTI-CD8a (#12-0081-81, Thermofisher), APC-ANTI-PD-1 (#17-9985-80, Thermofisher), APC-ANTI-TIM-3 (#17-5871-80, Thermofisher), APC-ANTI-BTLA (#17-5956-80, Thermofisher), FITC-ANTI-CD11c (#11-0114-82, Thermofisher), APC-ANTI-GALECTIN-9 (#17-9211-80, Thermofisher),

	PE-ANTI-PD-L1 (#12-5982-81, Thermofisher), APC-ANTI-CD25 (#17-0251-81, Thermofisher), PE-ANTI-FOXP3 (#12-5773-80, Thermofisher), APC-ANTI-TIM-3 (#17-5871-80, Thermofisher), ANTI-GALECTIN-9 (#Z38004, Baiao Leibo Biotechnology Co., Ltd)
Validation	Flow cytometry, western blot

Eukaryotic cell lines

Policy information about cell lines and Sex and Gender in Research

Cell line source(s)	A murine melanoma cell line B16-F10
Authentication	the American Type Culture Collection (ATCC)
Mycoplasma contamination	There are no phenomenon of mycoplasma contamination.
Commonly misidentified lines (See ICLAC register)	N/A

Animals and other research organisms

Policy information about studies involving animals; ARRIVE guidelines recommended for reporting animal research, and Sex and Gender in Research

Laboratory animals	Female 6 to 8-week-old C57BL/6 mice
Wild animals	N/A
Reporting on sex	N/A
Field-collected samples	N/A
Ethics oversight	Yes

Note that full information on the approval of the study protocol must also be provided in the manuscript.

Plants

Seed stocks	N/A
Novel plant genotypes	N/A
Authentication	N/A

ChIP-seq

Data deposition

☒ Confirm that both raw and final processed data have been deposited in a public database such as GEO.

☒ Confirm that you have deposited or provided access to graph files (e.g. BED files) for the called peaks.

Data access links *May remain private before publication.*	The Cancer Genome Altas database; The GEO database (GSE215120); http://ncbi.nlm.nih.gov/gene/
Files in database submission	.tsv; .mirnas.quantification; GSM6622299; GSM6622300; GSM6622301
Genome browser session (e.g. UCSC)	http://ncbi.nlm.nih.gov/gene/

Methodology

Replicates	3
Sequencing depth	single cell 3' v2 protocol, 10x Genomics

Antibodies	N/A
Peak calling parameters	HiSeq X Ten
Data quality	HiSeq X Ten
Software	Cell Ranger software v2.1.1

Flow Cytometry

Plots

Confirm that:

☒ The axis labels state the marker and fluorochrome used (e.g. CD4-FITC).

☒ The axis scales are clearly visible. Include numbers along axes only for bottom left plot of group (a 'group' is an analysis of identical markers).

☒ All plots are contour plots with outliers or pseudocolor plots.

☒ A numerical value for number of cells or percentage (with statistics) is provided.

Methodology

Sample preparation	B16-F10 or splenic lymphocyte suspension was incubated with monoclonal antibodies at 4 Celsius degree for 30 min, and then washed twice with PBS and resuspended with 300 microliter PBS for test.
Instrument	BD Bioscience
Software	CytoExpert 2.3
Cell population abundance	Gating 1000000 cells, and cell poplulation abundance is determined according to shift of fluorescence intensity.
Gating strategy	According to the references and protocols to gate cells.

☒ Tick this box to confirm that a figure exemplifying the gating strategy is provided in the Supplementary Information.
